# Dandelion‐Like CuWO_4_/WO_3_ Composite Photoanode Employing Layered Double Hydroxide Catalysts for Enhanced Photoelectrochemical Water Oxidation

**DOI:** 10.1002/cssc.202502130

**Published:** 2025-12-14

**Authors:** Sima Nouhi, Michael Wark, Dereje Hailu Taffa

**Affiliations:** ^1^ Institute of Chemistry Chemical Technology 1 Carl von Ossietzky University of Oldenburg Oldenburg Germany

**Keywords:** co‐catalyst, oxygen evolution reaction, photoanode, semiconductor, water splitting

## Abstract

This study introduces a novel modified dandelion‐like CuWO_4_/WO_3_ heterojunction photoanode featuring NiCo‐layered double hydroxide (NiCo‐LDH) as a co‐catalyst, fabricated through hydrothermal and drop casting methods. Morphological and photoelectrochemical assessments elucidate that the optimal dandelion‐like CuWO_4_/WO_3_ heterojunction facilitates charge separation in a neutral solution. Additionally, electrochemical evaluations of the CuWO_4_/WO_3_ heterojunction decorated with LDHs co‐catalysts reveal enhancements in photon‐to‐current conversion efficiency (IPCE), cathodic shift of the photocurrent onset potential, and an increase in photocurrent density to 0.17 mA cm^−2^ at 1.23 V vs. RHE, which is twice that of the pristine photoanode under neutral pH conditions. The improved performance of the modified photoanode is attributed to the accelerated diffusion of reactants and products, as well as efficient proton‐coupled electron transfer processes facilitated by the presence of LDHs.

## Graphical abstract

Hydrothermally grown WO_3_/CuWO_4_ composite photoanode with tunable dandelion‐like structure improves photocurrent response. The synthesis parameters control the size and distribution of the dandelion structures, there by governing the composition and photoelectrochemical activity.

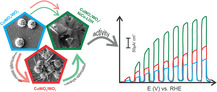

## 1 Introduction

In recent decades, the demand for alternative and renewable fuels has surged in response to the worldwide depletion of energy resources and the growing emphasis on environmental conservation. Hydrogen has emerged as a widely recognized candidate for future energy production due to its sustainability and environmentally benign characteristics [1]. One of the most promising avenues for directly converting solar energy into hydrogen is through photoelectrochemical (PEC) water splitting. This process relies on light‐absorbing semiconductors to drive two electrochemical half‐cell reactions: the hydrogen evolution reaction (HER) and the oxygen evolution reaction (OER). Despite offering an efficient means of producing high‐purity hydrogen, its widespread implementation for large‐scale production remains constrained. The OER, which is necessary for the process, poses greater challenges than the HER due to its requirement for transferring four electrons (2H_2_O → O_2_ + 4e^−^ + 4H^+^), leading to the reorganization of chemical bonds between water molecules. Consequently, there has been significant interest in designing efficient and cost‐effective catalysts for the PEC oxygen evolution reaction in recent years [2]. So far, various metal oxides semiconductors have been developed for PEC water splitting including TiO_2_ [3], ZnO [4], Fe_2_O_3_ [5, 6], and WO_3_ [7]. WO_3_ has garnered considerable attention, primarily due to its nontoxic composition, notable properties such as high hole mobility (∼10 cm^2^ V^−1^ s^−1^), strong resistance to photo corrosion, and stability in acidic solutions. Nonetheless, its relatively wide bandgap energy (2.7 eV) restricts its activity to the near UV‐blue visible region and a narrow pH range conducive to stable operation (pH < 5), thereby constraining its widespread application on a large scale [8, 9]. To address these limitations, a potential approach involves the synthesis of ternary and quaternary oxides. This entails the introduction of a second metal into binary oxides, aiming to narrow the band gap and elevate the valence band position [10]. Among the group of WO_3_‐based ternary oxides, CuWO_4_ stands out with its relatively smaller bandgap energy, approximately ranging from 2.25 to 2.45 eV [11, 12, 13, 14]. Therefore, it has the capability to capture a greater portion of the visible spectrum [15]. In addition, CuWO_4_ demonstrates improved chemical stability, light absorption, and selectivity for OER versus its binary oxide counterpart WO_3_ [11]. However, the sluggish charge separation within the electrode bulk and slow kinetics of surface water oxidation are factors contributing to the overall inefficiency of PEC water oxidation at CuWO_4_ [16, 17]. Consequently, considerable efforts have been directed toward developing the PEC performance of CuWO_4_, including fabrication of heterojunction through coupling with other semiconductors such as BiVO_4_ [18], Mn_3_O_4_ [19], WO_3_ [20], or carbon‐based compounds such as multiwall carbon nanotubes (MWCNT) [21], surface modification with plasmonic nanoparticles Au, Ag [2, 22], or with carbodiimides‐based compounds like Sn_2_O(NCN) [23], Ag_2_NCN [24], nitrogen or hydrogen gas treatment to create oxygen vacancies [25, 26], and doping with metallic elements [27, 28, 29, 30]. Among these different methods, incorporating oxygen‐evolution cocatalysts (OECs) has proven to be an efficient approach for accelerating surface reaction kinetics [31]. Phosphate‐based catalysts are among the well‐explored OECs for CuWO_4_ thin films. Research conducted by Chen et al. demonstrated that CuWO_4_ electrodes catalyzed by cobalt phosphate complexes exhibited an 86% increase in photocurrent response compared to unmodified CuWO_4_ nanoparticles [32]. Xiong et al. documented a notable increase in both photocurrent and incident photon to current efficiency (IPCE) for the water splitting reaction following the integration of nickel phosphate (Ni–Pi) nanoparticles onto the CuWO_4_ photoanode surface [33].

Layered double hydroxide (LDHs) stands out as a remarkable OEC for water oxidation. LDHs are structured as layered anionic clay materials, characterized by a general formula [M^II^_1‐x_ M^III^_x_ (OH)_2_]^x+^(A^n−^)_x/n_ .mH_2_O, in which M^II^ and M^III^ represent di‐ and trivalent metal cations, respectively, and A^n−^ denotes counter anions. This layered arrangement offers numerous advantages, including substantial surface‐to‐bulk ratios, more effective exposure of catalytic active sites compared to 0D and 1D materials, and hierarchical porosity that facilitates the diffusion of water molecules and release of gaseous products [34]. In photoelectrochemical reactions, LDHs are mainly used in two types of reactions, the conversion of organics to high value chemicals [35, 36] and water splitting reactions to store hydrogen [37, 38]. In the latter case, Yue et al. reported that super‐hydrophilic H‐CoAl‐LDH/BiVO_4_ photoanode yielded a photocurrent of 3.5 mA cm^−^^2^ at 1.23 V versus RHE, representing ≈3.2 times the photocurrent of a pure BiVO_4_ photoanode [37]. Likewise, Bai et al. demonstrated the photocurrent density of the WO_3_/Fe_2_O_3_ heterojunction decorated by NiFe‐ LDH reached up to 3.0 mA cm^−2^, which respectively were five times and seven times of WO_3_ and *α*‐Fe_2_O_3._ [39]

To the best of our knowledge, this study marks the first exploration of LDHs as catalyst on CuWO_4_/WO_3_ thin films for the oxygen‐evolving half‐reaction at neutral electrolyte. In the present study, the detailed growth mechanisms of dandelion‐like CuWO_4_/WO_3_ were investigated. The dandelion structure resembles flower‐like structures but features radially oriented spikes or rod‐like structures. This results in a more open architecture with a high degree of symmetry, which can enhance electrolyte penetration and light scattering expected to benefit PEC performances. Furthermore, enhanced PEC properties of the CuWO_4_‐NiCo LDHs photoanode and potential photo‐electrocatalytic mechanisms were discussed.

## 2 Experimental Section

### 2.1 Chemicals

The following analytical grade chemicals were purchased and used as supplied: ammonium metatungstate [(NH_4_)_6_H_2_W_12_O_40_] (Sigma–Aldrich), copper (II) chloride 99% anhydrate [CuCl_2_] (Alfa Aesar), nickel (II) nitrate hexahydrate [Ni(NO_3_)_2_.6H_2_O] (Alfa Aesar), cobalt (II) nitrate hexahydrate [Co(NO_3_)_2_.6H_2_O] (Alfa Aesar), ammonium fluoride [NH_4_F] (Sigma–Aldrich), urea [CO(NH_2_)_2_] (Sigma–Aldrich), monosodium phosphate [NaH_2_PO_4_] (Alfa Aesar), disodium hydrogen phosphate [Na_2_HPO_4_] (Alfa Aesar), sodium hydroxide [NaOH] (Alfa Aesar), phosphoric acid [H_3_PO_4_] (Fisher Chemical), sodium sulfate [Na_2_SO_4_] (Fisher Chemical), Sodium sulfite [Na_2_SO_3_)] (Fisher Chemical), Nafion (10% ethanolic solution, Sigma), and EtOH (Fisher Chemical). Double distilled water was used for the preparation of all solutions. Fluorine‐doped tin oxide (FTO)‐coated glass sheets (2.2 mm thickness, 15 Ω) were purchased from Pilkington Glass.

### 2.2 Preparation of CuWO_4_/WO_3_, NiCo‐LDH, and CuWO_4_/WO_3__4_/NiCo‐LDH

Here, CuWO_4_/WO_3_ photoanodes were prepared by simple, one‐pot hydrothermal method. The initial solution was prepared by dissolving 2 mmol of the respective precursors, ammonium metatungstate (NH_4_)_6_H_2_W_12_O_40_) and copper chloride dihydrate (CuCl_2_·2H_2_O) in deionized water and stirring for 1 h. Subsequently, equal volumes of the resultant solution was transferred into a 20 mL Teflon‐lined stainless‐steel autoclave with the clean conductive fluorine‐doped tin oxide (FTO) glass substrates immersed into the solution. In the final solutions, the metal ratios of Cu^2+^/W^6+^ is nearly 1:12. Accordingly, higher precursor concentrations were used while keeping the metal ratios the same. The reaction was carried out at 180°C for 4, 6, 8, 10, 12, and 14 h, respectively. Afterward, the autoclave was cooled to room temperature naturally. The obtained films were washed with ethanol and subsequently annealed in furnace at 500°C for 2 hr. The films were heated and cooled at a rate of ≈2°C/min. After annealing, the resulting bright yellow films were used in all subsequent experiments. NiCo‐LDH were synthesized through hydrothermal reaction as well. 0.5 mmol of Ni(NO_3_)_2_ · 6H_2_O, 1 mmol of Co(NO_3_)_2_·6H_2_O, 3 mmol of NH_4_F, 7.5 mmol of urea were dissolved in 30 mL of deionized water. After stirring for 30 min, the obtained light pink‐colored solution was poured into a 70 mL Teflon‐lined stainless‐steel autoclave and was kept at 120°C for 10 hr. After cooling to room temperature, the pink NiCo‐LDH powder was washed with ethanol and deionized water three times, respectively. For loading the co‐catalyst (NiCo‐LDH) onto a CuWO_4_/WO_3_ photoanodes, drop‐casting method was used. 2 mg of NiCo‐LDH nanoparticles was dispersed in a 2 mL nafion solution and sonicated for 30 min to obtain uniform catalyst “ink” solution. The area of the drop‐casted region was carefully regulated to 2 cm^2^, while the mass of NiCo‐LDH was controlled by the volume of ink used. Finally, the composites were dried at 25°C.

### 2.3 Characterization

#### 2.3.1 Physicochemical Characterization

Diffuse reflectance UV–VIS measurements were carried out using a Varian Cary 4000 in the range of 200–800 nm to find out about optical properties of the photoanodes. The surface composition and chemical state of the materials was examined by X‐ray photoelectron spectroscopy (XPS) measurements with a Thermo Fischer ESCALAB 250 Xi photoelectron spectrometer with a monochromic Al K*α* X‐ray source (1486.6 eV). The morphology and the elemental distribution of the samples were determined by scanning electron microscopy (SEM) and energy‐dispersive X‐ray spectroscopy measurements using an FEI Helios NanoLab 600i electron microscope. The crystallinity of the prepared catalysts was studied by X‐ray diffractograms (XRD) (PANalytical Netherlands, Empyrean Series 2) with Cu K*α* radiation (*λ* = 0.154 nm). Photoelectrochemical measurements were performed using a Zahner Zennium E potentiostat (Zahner Elektrik GmbH) running under Thales's software (version 4.12). Raman spectras were recorded by a Bruker Senterra Raman spectrometer using a 488 nm laser with 0.2 mW laser power. The Fourier transform infrared spectra (FT‐IR) were obtained using a Bruker FT‐IR Tensor 27 Spectrometer with a platinum ATR unit.

#### 2.3.2 Photoelectrochemical Measurements

Electrochemcial characterization was conducted in a specially designed cell in a three‐electrode configuration with the CuWO_4_ thin film as the working electrode, a Pt wire counter electrode, and an Ag/AgCl reference electrode. The actual geometric area of the working electrode with a A = 0.785 cm^2^ was exposed to front‐side illumination in the electrolyte (Na_2_SO_4_ 0.1 M in phosphate buffer solution PBS, pH = 7). For chopped light voltammetry (CLV) measurements, a white LED light source (1200 W·m^−2^ incident photon flux) controlled by PP211 potentiostat (Zahner–Elektrik GmbH) was used. The potential was scanned at a rate of 10 mV/s between 0 V and +1 V versus Ag/AgCl with light on and off cycles each lasting for 10 s. For comparison purposes, the applied potentials versus Ag/AgCl(sat.KCl) were converted into potentials relative to the reversible hydrogen electrode (RHE) by using the Nernst equation:

(1) $$E_{\mathrm{RHE}}=E_{\mathrm{Ag}/\mathrm{AgCl}\left(\mathrm{sat}.\mathrm{KCl}\right)}+0.0591\;pH+E_{\mathrm{Ag}/\mathrm{AgCl}\left(\mathrm{sat}.\mathrm{KCl}\right)}^{0}$$

Electrochemical impedance spectroscopy (EIS) was recorded within the frequency range 10 mHz to 100 kHz with 10 mV AC rms amplitude at 1.23 V versus RHE under the illumination. Mott−Schottky measurements were carried out in the above three electrode cell within the potential range of 0.3–1.4 V versus RHE in the dark at the frequency of 1000 Hz.

## 3 Results and Discussion

**FIGURE 1:**
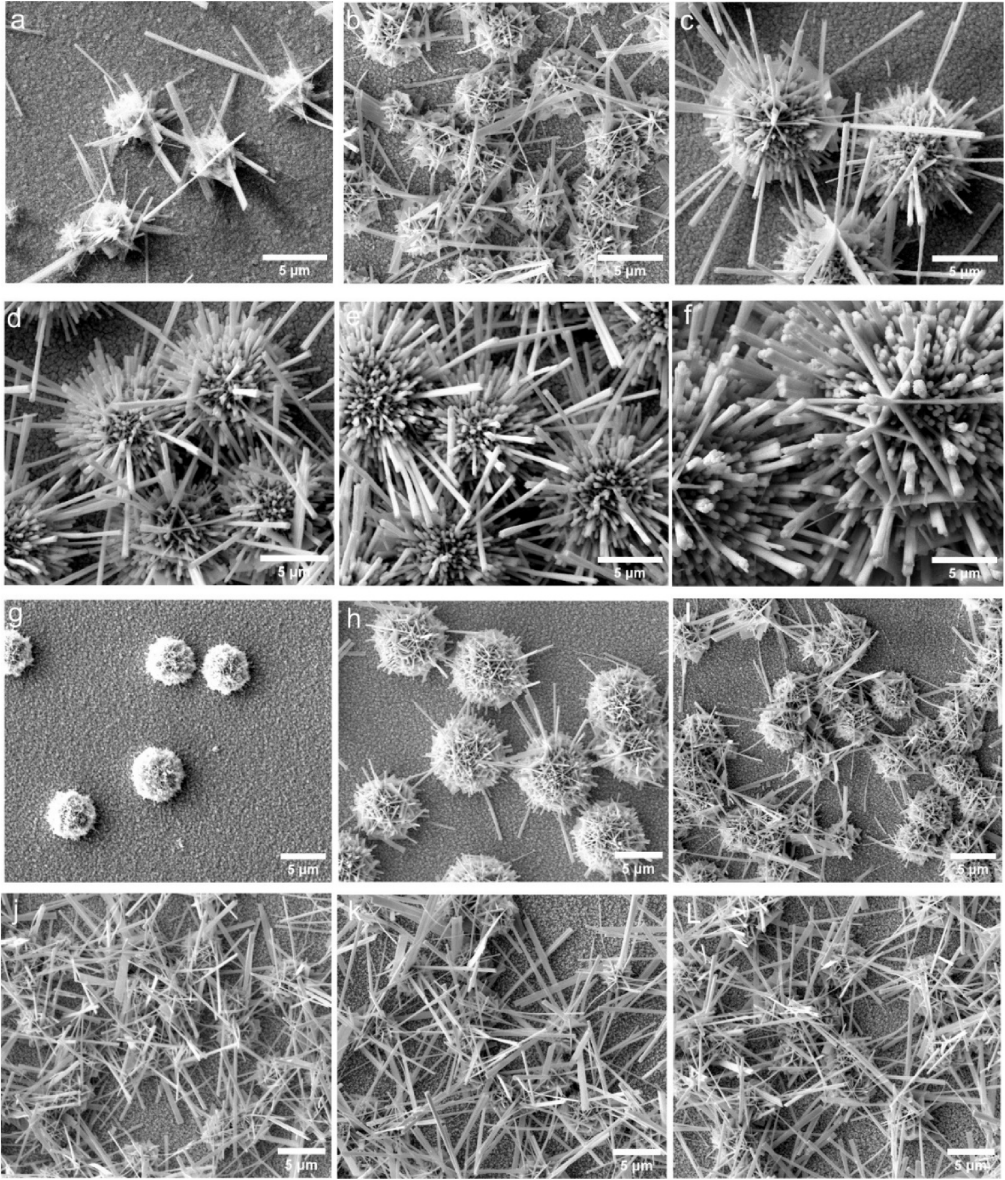
Top view SEM images of CuWO_4_/WO_3_ films showing the influence of different hydrothermal parameters: Influence of precursor concentration (mM) while keeping the T at 180°C and the synthesis time at 8h: (a) 1 mM, (b) 2 mM, (c) 3 mM, (d) 4 mM, (e) 5 mM, and (f) 10 mM; and the variations of temperature while maintaining the precursors concentration and synthesis time at 2 mM and at 8h, respectively: (g) 140°C, (h) 160°C, (i) 180°C, (j) 200°C, (k) 220°C, and (l) 240°C.

Via controlled hydrothermal reaction parameters including concentration, temperature, and time the morphological evolution and crystal growth mechanism of CWO thin films were evaluated. Figure 1 shows that the SEM images of the CWO films were synthesized by having a different precursors concentration. Initially, at low concentration of precursors, small and compact plate‐like and wire‐like structures were formed on a surface of uniform dense layer which composed of small grains on the FTO surface. By further concentration increase, new wire‐like structures with a growth orientation outward of the surface emerged. The wire‐like structures are well‐arranged in parallel way, consisting of several sub‐wires extending radially outward which created a dandelion‐like microsphere structure with average diameters of ca. 5 µm (Figure 1b). The further increasing of concentration impelled dandelion‐like structures with larger diameters around 35 µm with longer wires around them (Figure 1f). Subsequently, the effect of various reaction temperatures reveal that, increasing the temperature to 200°C and higher lead to the reduction of microspheres sizes with laterally grown elongated wire‐like structures covering the entire surface (Figure 1g,h). This phenomenon could be explained by the competition between nucleation and growth, at low temperature nucleation is favored resulting more microspheres but as temperature increases crystal growth is dominant and leading the formation of elongated wires. Moreover, by changing the reaction time, the coverage of dandelion structures on the surface were tuned, and the highest coverage was achieved after 8h Figure S1a–f. The lateral view and cross section SEM images of the CuWO_4_/WO_3_ film are presented in Figure S1g–h. The cross‐sectional view image reveals that a thin compact layer beneath the microspheres and the average thickness of the film is ≈300 nm, thereby affirming the film's formation on the surface of the FTO substrate. Additionally, the ratios of Cu:W were varied by modulating the precursor concentrations to have more insights to the morphological evolution of the composite films (Figure S2 a–c). The Cu:W ratios significantly affect the distribution of the microspheres on the surface. The surface is densely populated as the Cu:W ratio decreased from 1:6 to 0.25:6 (or as the amount of W increases). This partly suggests that the microspheres are mainly W rich phases. As we discuss in the later section and shown in Figure S2d, higher coverage of the microspheres is detrimental for the photocurrent response.

The growth mechanism underlying the formation of the dandelion‐like structure can be described as follows: During the initial phase of the hydrothermal reaction, nucleation occurred on the FTO substrate [14]. Subsequently, the growth of CWO crystals ensue from these nuclei. Considering the initial crystals possess thermodynamically unstable boundaries and grains, crystallization tends to progress in the most facile direction to minimize energy. Consequently, surface reconstruction further contributes to the formation of a dandelion‐like structure [10, 40]. Considering the surface coverage and morphological features (microspheres /wire hybrid structures), the optimal hydrothermal synthesis conditions are 180°C, and 8hr at precursor concentration of 2 mM. This sample was further investigated, and the results are compared with other sets of samples whenever necessary.

**FIGURE 2:**
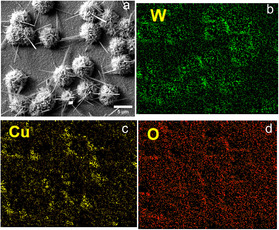
SEM image (a) and element distribution mapping of CuWO_4_/WO_3_ film with the optimal hydrothermal reaction conditions (2 mM, 8 h at 180°C). (b) W mapping, (c) Cu mapping, and (d) O mapping.

The elemental composition of optimal dandelion‐like CuWO_4_/WO_3_ thin film was analyzed by energy dispersive X‐ray spectrometer (EDS) (Figure 2).

The EDS analysis confirms the presence of Cu, W, and O elements within the thin film. The elemental mapping shows that W is predominantly found within the microspheres of the dandelion‐like structures, while Cu is primarily distributed on the flat sections of the film. The distribution of O is uniform throughout the film including the O contribution from the underlying FTO substrate. The EDS spectra, mass, and atomic percentages for all elements are demonstrated in Figure S1i. The atomic percentages of W and Cu indicated that the synthesized thin films possess a higher quantity of W (Cu:W, 1:2.6). This specific distribution pattern of Cu and W on FTO substrate corresponds to prior research finding. The Cu mostly distributes close to the FTO substrate while exhibiting a decrease in cohesion as it moves away from the substrate. Conversely, the W shows a greater distribution in the upper layers, further away from the substrate [41].

**FIGURE 3:**
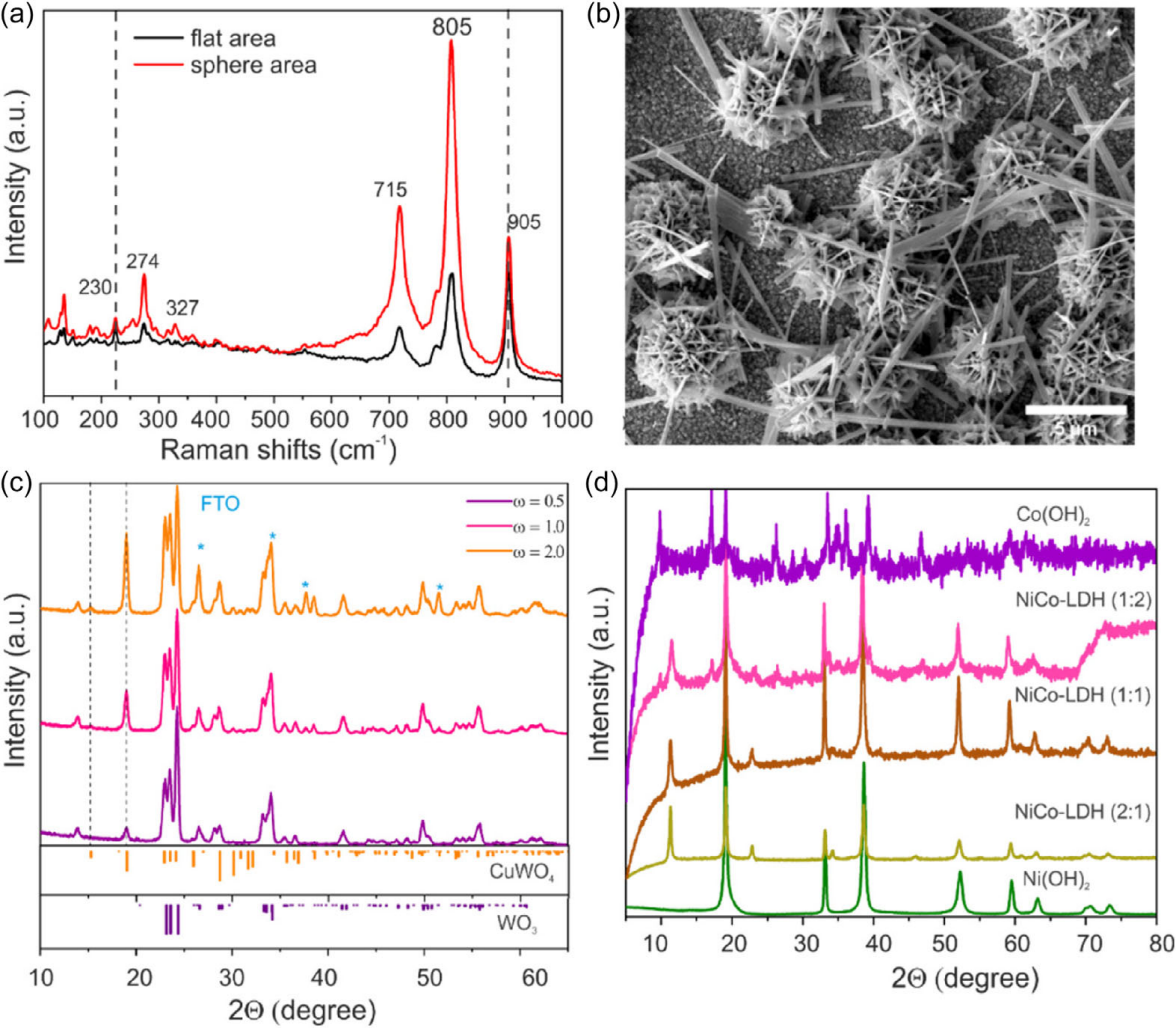
(a) Raman spectra, (b) SEM image, and (c) XRD pattern of the optimum CuWO_4_/WO_3_ composite film (2 mM, 8 h, 180°C) at different incident angles (*ω* = 0.5°, 1°. 2°). (d) XRD pattern of the catalyst NiCo‐LDH with various Ni/Co ratios of (1:0), (2:1), (1:1), (1:2), and (0:1).

Raman analyses were performed to further determine the surface property and phase information of the as prepared photoanode. The SEM images of CWO films show two distinct regions: dense flat surface and microsphere dandelion‐like structures on top. Site‐specific Raman spectra of the two different regions of the samples were recorded from samples prepared in the lowest and highest regimes of hydrothermal reaction parameters, e.g., concentration of precursors (1 mM and 10 mM), time of reaction (4 h and 14 h), and reaction temperatures (140°C and 240°C) were studied. The Raman spectra and corresponding SEM images are presented in Figure S3–S5. The Raman spectra of flat area of all samples demonstrate the sharp and intense vibrational Raman mode at around 905 cm^−1^ in agreement with triclinic structural CuWO_4_ reported in the literature [42]. This mode is attributed to the symmetric stretching mode of W‐O in (WO_6_) coordination environment [43]. Additionally, the Raman mode at 230 cm^−1^ is also assigned to one of the bending/rotational modes in CuWO_4_ which is typically absent in pure WO_3_ [44]. However, in the sample prepared at high temperature (240°C), the intensity of CuWO_4_ peak is decreased, and intense peaks at 805 and 715 cm^−1^ are visible, which are attributed to the stretching vibrations of bridging oxygen in O–W–O. This suggests that high hydrothermal temperature conditions favor the formation of WO_3_ phase. The Raman peaks at 274, 327, 715, and 805 cm^−1^ are assigned to monoclinic type WO_3_ structure. The peaks located in 327 and 274 cm^−1^ Raman mode are corresponding to bending vibration of O–W–O. Raman spectra of sphere area of all the samples clearly exhibit the intense peaks of monoclinic type WO_3_ structure. For samples prepared at high concentration and high temperature, a wide shoulder in the 590–690 cm^−1^ region may result from the transition between different WO_3_ crystallographic phases. Moreover, there is a peak at the lower wavenumber of 140 cm^−1^ which can be attributed to lattice modes [41, 45]. Figure 3a,b depicts the Raman spectra and SEM image of the optimum CuWO_4_ sample, respectively. The Raman analysis reveals that as‐prepared photoanodes are the composite of CuWO_4_/WO_3_ with a structure consisting of CuWO_4_ as a dense homogenous layer on surface of FTO and dandelion‐like microsphere of WO_3_ rich upper layer.

X‐ray diffraction patterns reveal material structures because of its quantitative and nondestructive analysis features. In the case of thin films, grazing geometry is required to increase the interaction of X‐rays with the materials in the films. In this method, the information is obtained from the planes perpendicular to the surface. As a result, the adequate depth of penetration of X‐rays perpendicular to the surface decreases as the angle of incidence decreases up to the critical angle limit at which total reflection occurs [46]. Figure 3c displays Grazing incidence X‐ray diffraction (GIXRD) patterns of the optimum CuWO_4_/WO_3_ (2 mM, 8h and 180°C) film which were measured at different incident angles of 0.5°, 1°, 2°. At a low incident angle of 0.5°, the X‐ray penetrates just a few nanometers deep into the film. Thus, this diffraction pattern reflects the structure of the surface layer only, which indicates the main diffraction peaks of monoclinic WO_3_ structures at 2*θ* angles of 23.1, 23.6, and 24.4 corresponding to (002), (020), and (200) plane. The low‐intensity diffraction peak at 2*θ* = 19.0° refers to the (100) plane of triclinic CuWO_4_. When the incidence angle is increased to 1° or 2°, there is a significant intensity change of the (100) diffraction peak and appearance of new diffraction peak (010) at 2*θ* = 15.2° which is assigned to triclinic structure of CuWO_4_. This intensity alterations signify that more CuWO_4_ localized near the surface of FTO. When the incident angle was at 0.5°, the X‐ray probes the top microsphere layer which is dominantly the monoclinic WO_3_ phase, and at higher angles, the X‐ray reaches the flat dense layer of triclinic CuWO_4_. It can be concluded from these observations that there are two distinct phases present on the FTO surface. During the initial stages of film growth, CuWO_4_ was deposited on the surface, and, with further growth of the film, WO_3_ gradually accumulated on the CuWO_4_ layer. This finding is consistent with the results obtained from Raman and EDX measurements. Based on the Raman and XRD results, the growth of the composite films can be summarized as follows. Considering the acidic nature of the starting solution mixture (metatungtstate and copper chloride), the nucleation and growth WO_3_ is initially favorable, but the diffusion of Cu^2+^ ions to the growing WO_3_ crystals leads to the formation of CuWO_4_ under the hydrothermal conditions. As the reaction progresses, the Cu^2+^ ions are depleted from the solution, and the dynamic equilibrium of NH^4+^/NH_3_ species slightly increases the pH. The increase in pH leads to a competition between the growth and dissolution of WO_3_ rich microspheres resulting in corrugated microspheres.

Further, the prepared NiCo‐LDH co‐catalysts with varying Ni/Co ratios were investigated by XRD to identify the structures and phases (Figure 3d). The XRD patterns of the NiCo‐LDH with different ratios show reflexes at 2*θ* degree of 11.3°, 23.0°, and 34.3° are attributed to the (003), (006), and (009) planes of the hydrotalcite‐structured NiCo‐LDH. [47, 48] Conversely, the pure Ni (OH)_2_sample exhibited intense and sharp diffraction peaks of (001), (100), (101), and (102) planes at 2*θ* values of 19°, 33°, 38°, and 51°, respectively, assigned to the pure β‐Ni(OH)_2_ phase [49]. As‐prepared Co(OH)_2_‐ exhibited diffraction patterns of *α*‐hydroxides with low crystallinity, characterized by relatively weak diffraction peaks at 25°, 33.69°, and 37.96° which correspond to the (006), (012), and (015) planes [47, 50]. Generally, the NiCo‐LDH exhibits two distinct phases, namely the α‐phase and the β‐phase. The β‐phase is thermodynamically stable, and it is attributed to the hexagonal close‐packed structure of M^2+^ ions (M = Ni, Co) and OH^−^ ions, which results in higher crystalline structure with an interlayer spacing of ≈0.46 nm. On the other hand, the α‐phase is composed of M(OH)_2_ interlayers (M = Ni, Co) with low crystallinity and an interlayer spacing ranging from 0.75 to 0.81 nm [51]. Hence, the XRD results reveal that the NiCo‐LDH possesses crystalline features of α‐phase Co(OH)_2_ and β‐phase Ni(OH)_2_.

Additionally, the functional groups of the prepared LDH samples were determined by means of Fourier transform infrared (FT‐IR) spectrometer (Figure S6). The FT‐IR spectra of NiCo‐LDH samples with different mass ratio of Ni^2+^ and Co^2+^ in the region between 500 and 4000 cm^−1^, illustrated in Figure S5. The sharp peak centered at 3626 cm^−1^ is originated from free ‐OH groups of the typical β‐phase hydroxides [52]. The bands at 3525 and 1630 cm^−1^ are assigned to the O–H stretching vibration and bending modes of the interlayer water and hydroxyl group, respectively [53, 54]. Another band around 1383 cm^−1^ is indexed to the vibration of interlayer CO_3_^2−^ and NO_3_^2−^ anions. Atmospheric CO_2_ may adsorb on to the LDH, forming surface carbonate hydroxide (CO_3_^2−^ ions) species via coordinative bonds with Ni^2+^ and Co^2+^ ions. On the other hand, the NO_3_^−^ ions originating from the metal precursors are retained in the interlayer of LDH as counter ions [47]. The peaks in the region 500–700 cm^−1^ corresponds to metal–oxide, oxo‐metal‐oxo, metal‐oxo–metal bond, and bending vibrations [55].

**FIGURE 4:**
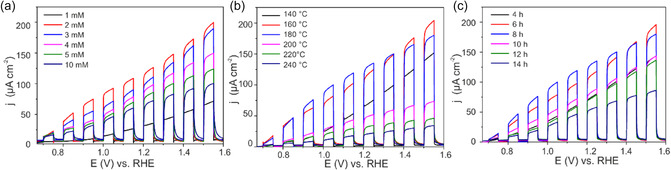
Influence of varied hydrothermal conditions on CLV of CuWO_4_/WO_3_ thin films: (a) precursor concentrations, (b) temperature, and (c) synthesis time.

The photoelectrochemical (PEC) response of the CuWO_4_/WO_3_ thin films were assessed by measuring the chopped light voltammetry of the films in 0.1 M Na_2_SO_4_ in phosphate buffer solution (PBS) pH = 7 (Figure 4). Figure 4a–c show the effect of the varied hydrothermal reaction conditions, including different precursor concentrations, temperatures, and reaction durations, on the CLV behavior of the films. The analyses revealed that the highest photocurrent was achieved with CuWO_4_/WO_3_ thin films synthesized using a precursor concentration of 2 mM, at a hydrothermal reaction temperature of 180°C for a duration of 8 h. The variation in photoelectrochemical performance was linked to notable alteration in the morphology of the composite microstructure, as confirmed by SEM observations (Figure 1). The highest activity was observed when the dandelion‐like microstructure of the CuWO_4_/WO_3_ composite was uniformly spread across the substrate, providing adequate active sites for surface reactions. It has been determined that achieving complete coverage, at high precursor concentration, leads to the formation of larger microspheres which obstruct the light path (due to shadowing effect) to the underling film resulting in low photocurrent response. On the other hand, at low concentration, film coverage is poor, and this reduces the number of available active sites for photoelectrochemical reactions at the electrode/electrolyte interface, leading to a diminished photocurrent response. Similarly, the presence of randomly scattered microspheres with substantial gaps on the photoanode's surface diminishes photoactivity likely due to the lack of light scattering effects. It has been shown that photoanodes with various morphologies, but similar chemistry shows varied photo current responses due to multiple scattering which led to higher light absorption [56]. By extending the reaction time, mainly the underlying compact film thickness increased without significantly affecting the microspheres sizes, and a thick film could potentially hinder the transport of charge carriers, resulting in an elevated rate of recombination. For vapor phase deposited films, it was demonstrated that under front side illumination, thicker CuWO_4_ films show low photocurrents, due to bulk electron–hole recombination [17, 44]. These results suggest that the number, size, and distribution of the microspheres should be optimized for higher photocurrent response.

**FIGURE 5:**
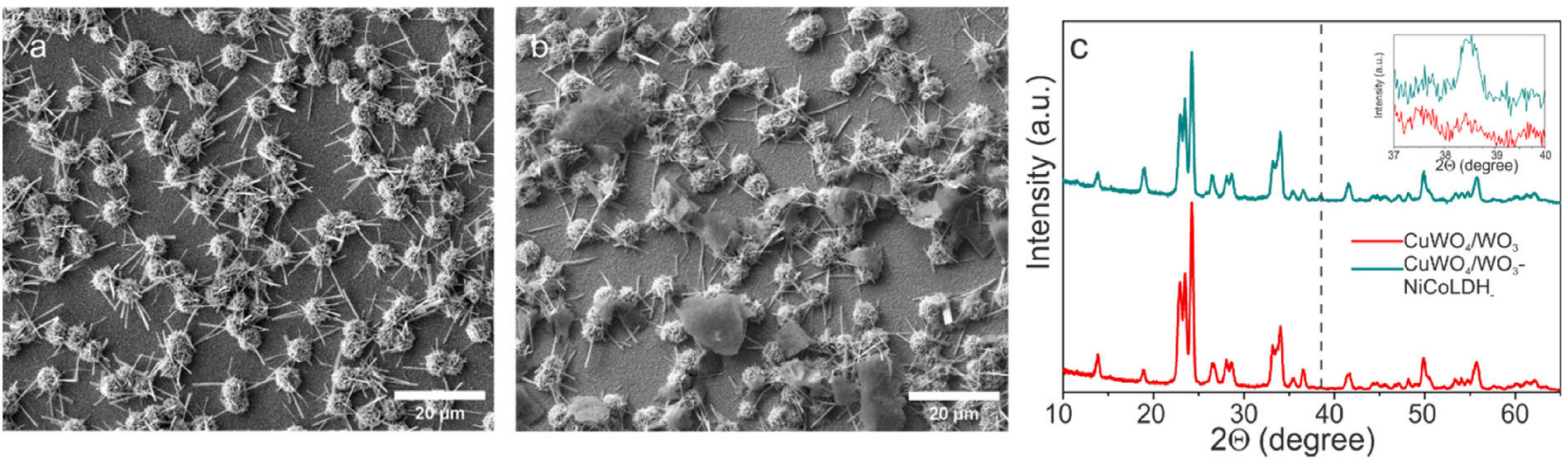
Top view SEM images of (a) CuWO_4_/WO_3_ film, (b) decorated CuWO_4_/WO_3_ film with NiCo‐LDH, and (c) GIXRD pattern of CuWO_4_/WO_3_ photoanode and modified one with NiCo‐LDH at the incident angle of 0.5°.

To improve the photocurrent response of the optimized photoanode NiCo‐LDH OER catalyst were prepared as described in the experimental section. The CuWO_4_/WO_3_ films were drop casted with solution containing different amounts of NiCo‐LDH. As shown in the SEM images (Figure 5b), the NiCo‐LDH (Ni:Co)(1:2) catalyst forms islands rather than a layer over the CuWO_4_/WO_3_ 3D dandelion‐like microstructures on the FTO substrate. The composition of modified film with NiCo‐LDH was further confirmed by the EDX mapping analysis (Figure S7) which clearly indicated the presence of all expected elements in the corresponding films, particularly cobalt and nickel as catalysts that were uniformly available within the LDH stacks. Furthermore, the GIXRD pattern of CuWO_4_/WO_3_ film shows a noticeable change after the deposition of NiCo‐LDH (Ni:Co)(1:2) LDH catalyst (Figure 5c) is the intensity increasing of CuWO_4_/WO_3_ film's diffraction peaks around 2*θ* at 19° and 34° which are corresponding to the sharp and symmetrical diffraction peaks of NiCo‐LDH catalyst, originating from β‐Ni(OH)_2_ phase in NiCo‐LDH.

Additionally, a new peak with 2*θ* value at 38.5° appears, referring to the (101) reflection of Ni(OH)_2_. The in set XRD patterns in the region of 37°–40° show no other impurity peaks, indicating that the LDHs have been of pure phases and the crystal structure of CuWO_4_/WO_3_ remains unaltered by the deposition of LDHs.

**FIGURE 6:**
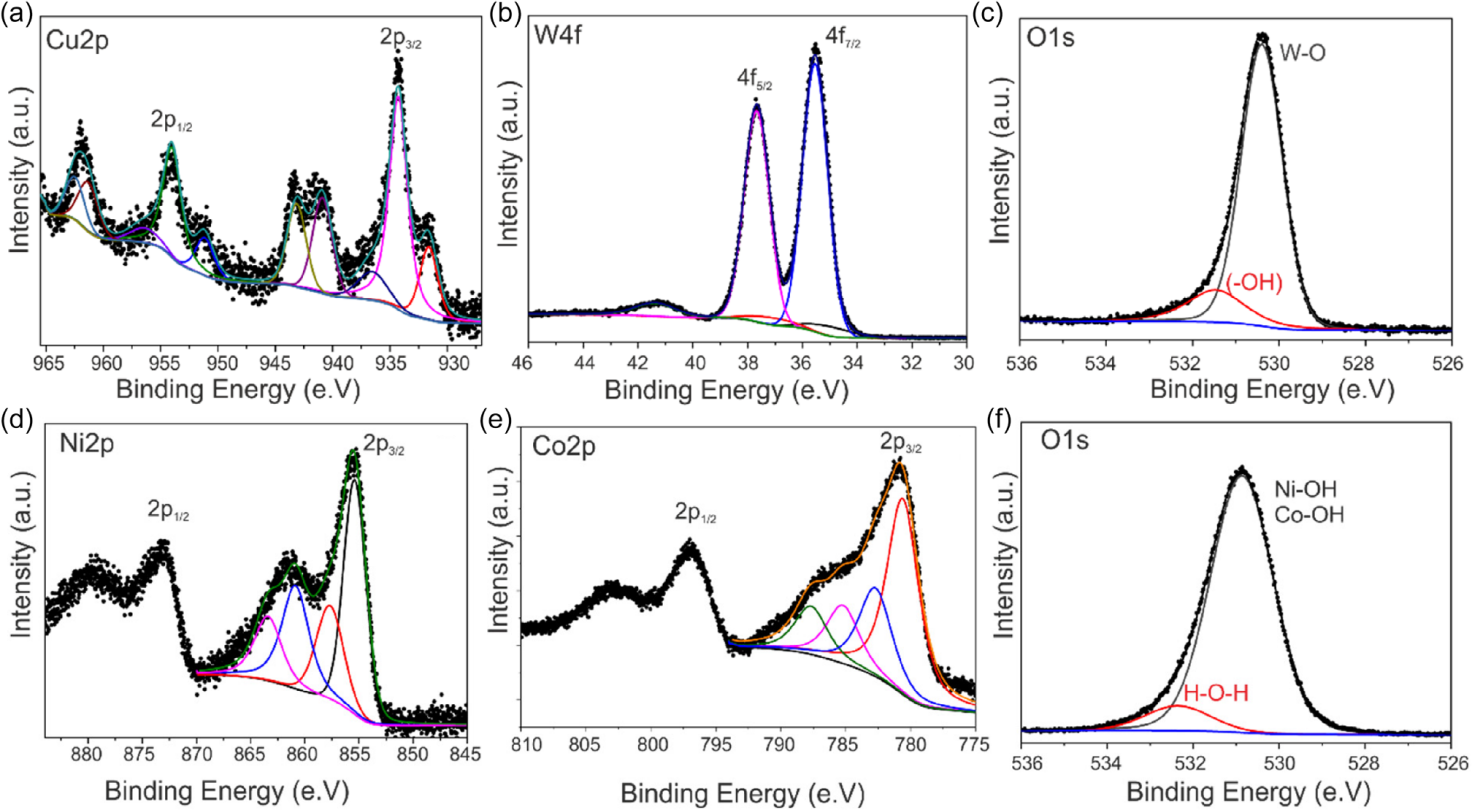
High resolution XP‐spectra of CuWO_4_/WO_3_ composite film (a) Cu 2p, (b) W 4f, and (c) O 1s; XP spectra of NiCo‐LDH (d) Ni 2p, (e) Co 2p, and (f) O 1s.

XPS technique was employed to confirm the surface modification and investigate the surface chemical states and elemental composition of the modified thin films. The core‐level Cu 2p XPS displays that Cu 2p_3/2_ and Cu 2p_1/2_ peaks are centered at the binding energies of 934.6 and 954.4 eV, which are the characteristic peaks of Cu^2+^ in the lattice sites of CuWO_4_ (Figure 6a) [26]. The high‐resolution W 4f XPS spectra can be deconvoluted into one pair of W 4f_7/2_ and W 4f_5/2_ peaks with the binding energies at 35.4 and 37.6 eV, respectively (Figure 6b). The peak energies are assigned to W^6+^ standard peaks. The high‐resolution spectrum of O1s (Figure 6c) consists of two peaks at 530.1 and 531.3 eV which are assigned to the lattice oxide, and surface hydroxide, respectively [41].

Figure 6a–c shows the XP spectra of the optimal LDH catalyst for CuWO_4_/WO_3_ photoanode prepared with with the (Ni:Co)(1:2) ratios (see below). The high‐resolution Ni 2p spectrum presents the individual peaks of Ni 2p_3/2_ and Ni 2p_1/2_ at the binding energy of 855.4 eV and 873.5 eV, accompanied with two main satellites peaks at 861.9 and 880.1 eV which demonstrate that the oxidation state of Ni is 2+ (Figure 6d) [57, 58]. The Co 2p core‐level spectrum(Figure 6e) displays the two major peaks around 781.6 eV and 797.0 eV which are in accordance with Co 2p_3/2_ and Co 2p_1/2_, along with two principal shakeup satellite peaks at 803.2 and 786.8 eV, which are an indicative of a high‐spin Co^2+^ in as‐prepared NiCo‐LDH [59, 60]. The O1s region can be deconvoluted into two peaks at 531.1 and 532.4 eV, which confirmed the presence of hydroxyl groups (M–OH) and oxygen bonds of H_2_O (H–O–H), respectively (Figure 6f) [61].

The XPS and EDX analyses validate that the atomic ratio of Ni to Co in the cocatalyst content is ≈1:2, which is highly consistent with the intended loading values (Table S1). These findings collectively demonstrate the successful formation of the CuWO_4_/WO_3_/NiCo‐LDH composite.

**FIGURE 7:**
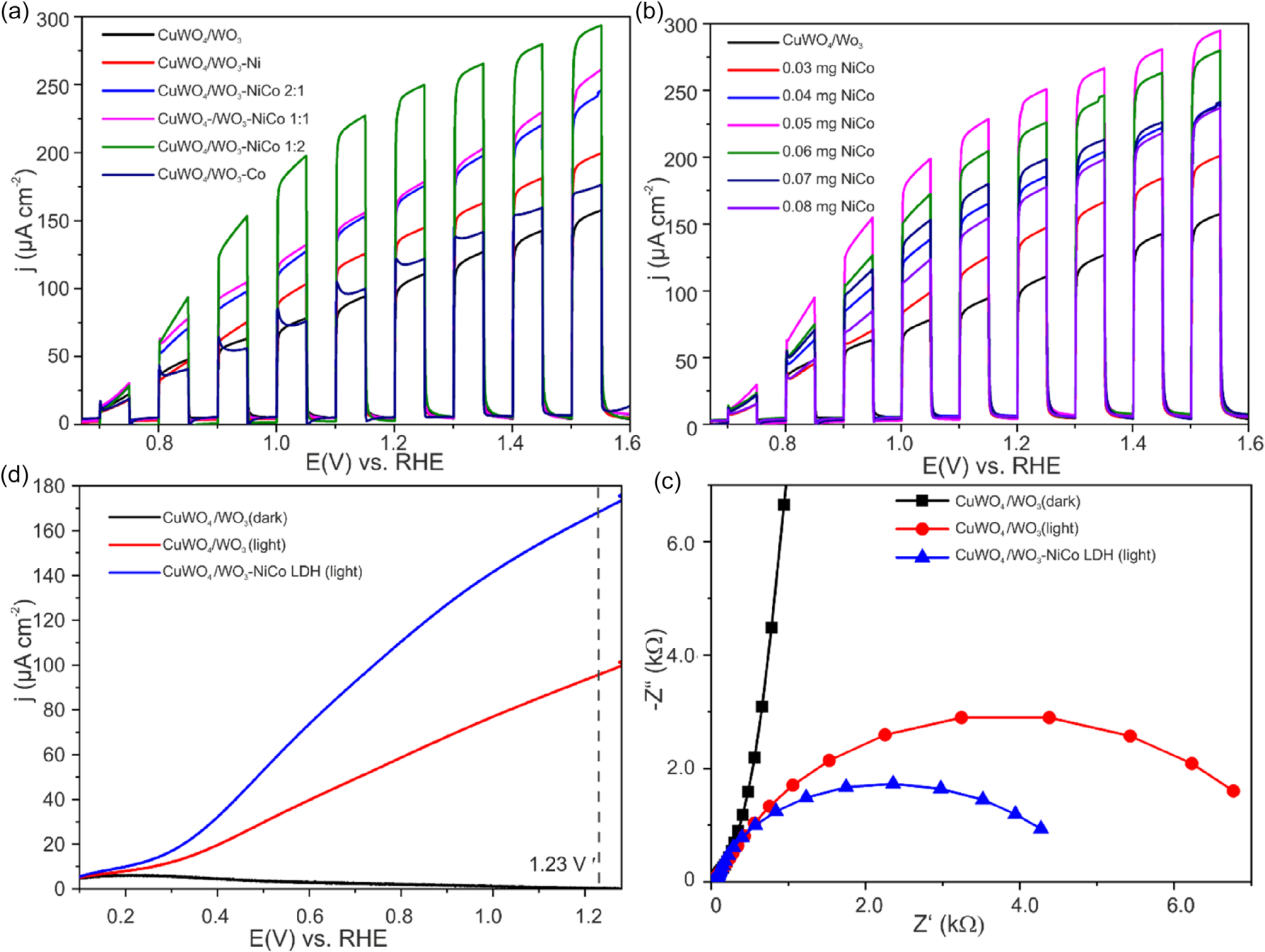
Chopped light linear sweep voltammogram of modified CuWO_4_/WO_3_ (a) showing the various ratio between (Ni:Co) at 0.05 mg LDH loading, (b) the influence of the of amount NiCo‐LDH for the optimized (Ni:Co) (1:2) ratio; (c) linear sweep voltammogram dark versus illumination, and (d) EIS Nyquist plot of CuWO_4_/WO_3_ photoanode and modified with NiCo‐LDH at 1.23 V versus RHE.

The catalyst coating should have no or minimal effect on the optical characteristics of CuWO_4_/WO_3_ films to have unhindered light penetration. To cross‐check this, the modified CuWO_4_/WO_3_ films were evaluated by means of UV‐Vis diffuse reflectance absorption spectra, as depicted in Figure 7Sa–b. Evidently, the absorption edge of CuWO_4_/WO_3_ bare film is roughly 520 nm, while the Tauc formula calculated bandgap is 2.30 eV, in good agreement with literature report [11, 62]. Accordingly, a suitable photo‐electrocatalyst should be as optically transparent as possible in the UV and visible‐light regions. The results show that the light absorbance of the modified film with NiCo‐LDH is slightly greater than that of the CuWO_4_/WO_3_ film; the absorption edge and Tauc plot of the modified film indicate nearly identical values. This implies that the loading of a LDH has a negligible impact on both light absorption and the electronic states of CuWO_4_/WO_3_ photoanode. The observation testifies that synthesized NiCo‐LDH with its weak light absorption capacity is an ideal OER cocatalyst candidate for photoanodes [8, 32].

The effect of LDH catalyst loading on the photoelectroactivity of CuWO_4_/WO_3_ semiconductor were explored by a series of PEC measurements. Figure 7a displays the PEC performance of the modified CuWO_4_/WO_3_ photoanode with NiCo‐LDH prepared with various ratio of Ni and Co. The highest activity assigned to NiCo‐LDH with a ratio (Ni:Co) (1:2). Considering the higher concentration of Co in the mixture (Ni:Co) (1:2), the prevalent phase is α‐Co(OH)_2_ (see above). The inherently wider interlayer spacing (≈0.75–0.81 nm) of the *α*‐LDH nanostructures, compared with ≈0.46 nm of β‐phase hydroxides, facilitates rapid and efficient access of electrolyte ions and water molecules to the active sites. Thus, these interlayer regions can serve as reservoirs for ion buffering in the aqueous electrolyte, thereby accelerating the electrochemical reactions. Our observations are consistent with earlier reports suggesting that Co‐rich cocatalysts enhance the performance of CuWO_4_ [17]. The loading of the catalyst further optimized for the most active LDH and CLV assessment shows that in comparison to the pure CuWO_4_/WO_3_ photoanode, the photocurrent density of the CuWO_4_/WO_3_/NiCo‐LDH composite photoanode improved with the increased loading of co‐catalysts and up to a catalyst loading of 0.05 mg of NiCo‐LDH (Figure 7b). Higher amounts of loadings result in reduced photocurrent which could be attributed to optical screening or unstable co catalyst layer.

The findings of the study indicate that the photocurrent density of the modified CuWO_4_/WO_3_ photoanode is significantly higher than that of the pristine CuWO_4_/WO_3_ photoanode as demonstrated by the linear sweep voltammograms (LSV) (Figure 7c). At 1.23 V versus RHE, the photocurrent density reaches 0.17 mA·cm^−2^, which is approximately twice the value of the pristine photoanode. The enhanced photocurrent density of CuWO_4_/WO_3_ electrodes is likely due to the efficient transfer of photogenerated holes from the valence states of irradiated CuWO_4_/WO_3_ to the NiCo‐LDH catalysts. This process effectively separates the photogenerated holes and electrons, thereby reducing interfacial charge recombination and ultimately enhancing the overall photoactivity [39, 63]. At the co‐catalyst layer the photogenerated holes could potentially oxidize the Ni^2+^/Co^2+^ species into their high‐valence states of Ni^3+^/Co^3^. At standard conditions (25°C and pH = 7), the redox potential for the Ni^ii^(OH)_2_/Ni^iii^OOH and Co^ii^(OH)_2_/Co^iii^OOH has been reported to be 1.4 and 1.2 V *vs*. RHE, respectively [64, 65]. Consequently, the generated Ni^3+^/Co^3+^ can oxidize the water molecules into O_2_ since the water oxidation potential is well below these oxidation potentials [33, 66].

To qualitatively assess the properties of charge transport between the electrodes and the electrolyte, photoelectrochemical EIS was measured at 1.23 V versus RHE. The typical Nyquist plot in Figure 7d shows a semicircle which represents the charge transfer resistance for the OER at the electrode/electrolyte interface [67]. The diameter of the semicircle (charge transfer resistance) decreases when the CuWO_4_/WO_3_ photoanode is modified with the co‐catalyst, providing clear evidence that electron transfer was facilitated in the presence of the catalyst.

**FIGURE 8:**
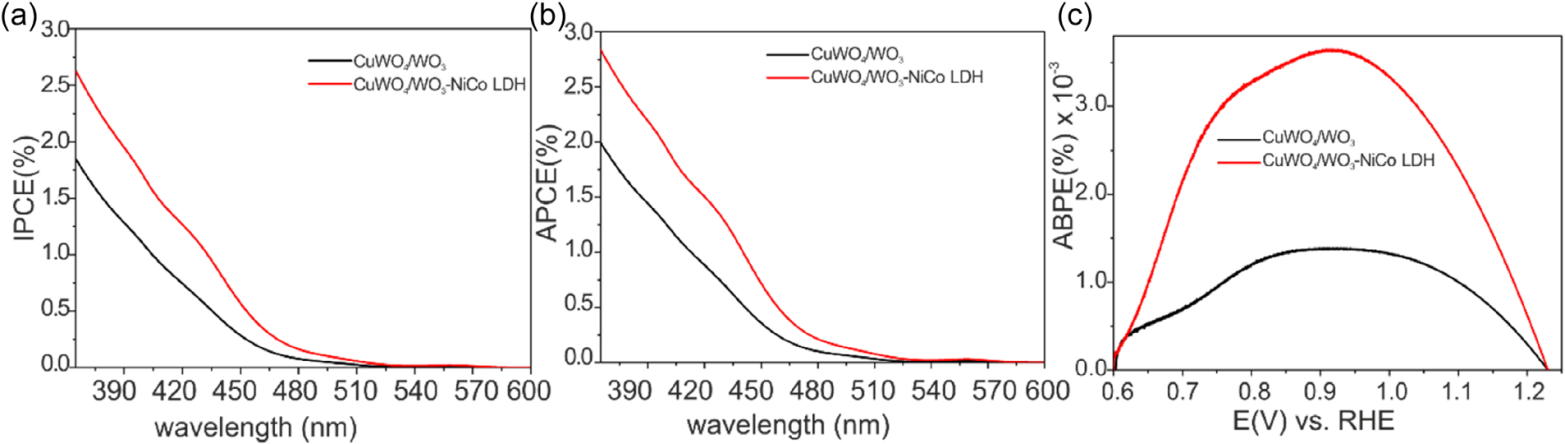
Influence NiCo‐LDH on the photoelectrochemical performance metrics of CuWO_4_/WO_3_ photoanode: (a) IPCE, (b) APCE, and (c) ABPE.

To evaluate how efficiently the absorbed photons are utilized to generate electric currents, the incident photons to current conversion efficiency (IPCE) and absorbed photons to current conversion efficiency (APCE) were evaluated via measurements taken at varying wavelengths at an applied bias of 1.23 V versus RHE (Figure 8a,b).

The values of IPCE are obtained using the following equation:

(2) $$\text{IPCE\%}=\frac{I_{\text{light}}\times 1240}{\lambda \times J}\times 100$$

where *J* is the photocurrent density, *I*_light_ is the incident light irradiance, and *λ* is the incident light wavelength. The absorbed photo‐to‐current efficiency (APCE) was acquired by dividing the IPCE by light harvesting efficiency (LHE) at each wavelength using the formula with

(3) $$\text{APCE\%}=\frac{\mathrm{IPCE}}{\mathrm{LHE}}\times 100$$

The LHE can be evaluated via equation:

(4) $$\mathrm{LHE}=1-10^{-A_{\lambda }}$$

where A is absorbance and *λ* is wavelength. With the modification of the NiCo‐LDH catalyst in the modified photoanode, both IPCE and APCE values were significantly improved. These findings demonstrate that the NiCo‐LDH effectively captures holes and leads to enhanced electron flow to the FTO back contact, ultimately resulting in higher photocurrent. The spectral resemblance of both IPCE and APCE with the absorption spectra (Figure S7a) suggests that there are no major losses during charge separation and no other parasitic absorption processes. However, the low values at specific wavelength are mainly attributed to the slow OER kinetics. Additionally, the electrode geometry, film thickness, and light illumination direction have significant influence on the measured values and need further optimization.

Another performance metric for single photoanode evaluation is the applied bias photon to current conversion efficiency (ABPE) is given by:

(5) $$\text{ABPE\%}=\frac{J_{\mathrm{PEC}}\times \left(1.23-V_{\mathrm{app}}\right)}{P_{\text{light}}}\times 100$$

where *J*_PEC_ is the photocurrent density (mA/cm^2^), *V*_app_ is the applied bias between the working electrode and counter electrode (V), and *P*_light_ is the incident illumination power density (AM 1.5G, 100 mW/cm^2^). The outcomes of ABPE reveal that the NiCo‐LDH catalyst increased efficiency by threefold; however, the value is well below 1%. This notably suggests the LDH catalyst is efficacious in separating charge carriers and enhancing the photoelectrochemical (PEC) capability of CuWO_4_/WO_3_ photoelectrodes in the absence of a hole scavenger (Figure 8c).

**FIGURE 9:**
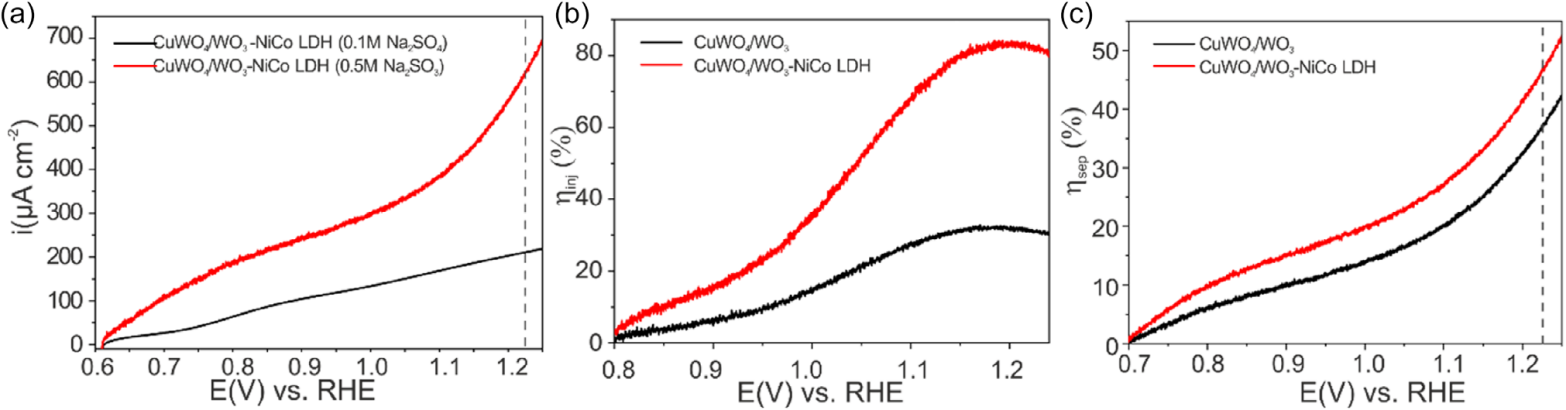
(a) J−V curve of CuWO_4_/WO_3_‐NiCo‐LDH photoanode in 0.5 M Na_2_SO_3_ and 0.1M Na_2_SO4 solution, (b) charge injection efficiency, and (c) charge separation efficiency.

The evaluation of the photoelectrode's inherent performance in terms of charge separation efficiencies and interfacial charge injection is often achieved through J–V measurements using hole and electron acceptors that possess fast interfacial charge transfer kinetics. Compounds which readily oxidize by photogenerated holes include sulfites and hydrogen peroxide. For this investigation, we utilized Na_2_SO_3_, a well‐known hole scavenger that exhibits the ability to rapidly consume photogenerated holes and oxidize itself to Na_2_SO_4_. [68, 69] As can be seen in Figure 9a, the water oxidation photocurrent (*J*_OER_) of CuWO_4_/WO_3_/NiCo‐LDH is lower than that of sulfite oxidation (*J*_sulfite_), demonstrating that the slow oxidation kinetics of water at the electrode/electrolyte interface was the primary cause of the loss of surface‐reaching holes. To quantify the contribution of NiCo‐LDH in improvement of surface charge transfer, namely the surface water oxidation reaction kinetics, the charge injection yield (*η*_injection_), which is the yield of holes that have reached the electrode/electrolyte interface and that are injected into the electrolyte to oxidize the water, was calculated using equation:

(6) $$\eta_{\text{injection}}=\frac{J_{\mathrm{PEC}}}{J_{\text{sulfite}}}$$

where *J*_PEC_ is the water oxidation photocurrent density in 0.1 M Na_2_SO_4_ (PH = 7 PBS) and *J*_sulfite_ is the photocurrent density in the presence of the hole scavenger (0.5 M Na_2_SO_3_) in the PBS electrolyte. The charge separation efficiency (*η*_sep_) on the surface of CuWO_4_/WO_3_/NiCo‐LDH photoanode can be obtained by dividing the photocurrent measured in the electrolyte containing Na_2_SO_3_ as a hole traper by the maximum theoretical photocurrentcurrent (*J*_abs_) (Equation (7)).

(7) $$\eta_{\mathrm{sep}}=\frac{J_{\text{sulfite}}}{J_{\mathrm{abs}}}$$

Figure 9b,c shows the calculated *η*_inj_ and *η*_sep_ curves for the photoanodes versus the applied potential. At 1.23 V versus RHE, the *η*_inj_ for CuWO_4_/WO_3_/NiCo‐LDH reached ≈82%, which is almost three‐fold higher than that of the CuWO_4_/WO_3_ photoanode (29%). It demonstrated that the NiCo‐LDH played a key role in accelerating the surface reaction by efficiently and quickly consuming photogenerated holes. Accordingly, the obtained *η*_sep_ values for CuWO_4_/WO_3_/NiCo‐LDH photoelectrodes are higher than to that of pristine CuWO_4_/WO_3_, which suggest the better charge separation efficiency of modified samples with NiCo‐LDH catalyst. These results indicate that loading NiCo‐LDH catalyst can significantly improve the separation and transfer of the photogenerated charge carriers.

Based on these results, the NiCo‐LDH catalyst used in this study can promote the separation of photogenerated charge carriers through consuming holes that accumulate on the photoanode electrolyte interface and accelerating charge transfer.

To investigate the stability of the LDH modified CuWO_4_/WO_3_ photohoanode, chronoamperometric measurements under chopped light illumination was performed for more than 2 h. The results in Figure S9 show that the photocurrent measured at 1.6 V versus RHE decreased from an initial value of 0.25 mA cm^−2^ to 0.2 mA cm^−2^ representing a 20% reduction. This could be attributed to the detachment of the LDH coating under the testing conditions. On the other hand, the photocurrent on the unmodified CuWO_4_/WO_3_ remains nearly constant. Further XPS investigations before and after the stability tests (Figure S10) reveal that, after the PEC test, no higher oxidation states of Cu and W is observed, but qualitatively, the signal intensities of Cu2p and W4f increased, suggesting that the outer LHD layer is probably partially removed (Figure S10 a,b). On the contrary, the binding energies of the Ni2p and Co2p are slightly shifted to higher binding energies (Figure S10c,d) indicating the oxidation of the LDH by the photogenerated holes supporting the hypothesis that Ni^3+^/Co^3+^ in the LDH are responsible for the higher OER currents.

Table S2 provides the comparison of PEC performance of reported data in literature for solution processed CuWO_4_ thin films for photoelectrochemical oxygen evolution reaction (OER). The recorded values for photocurrent density of pristine samples and modified ones are comparable with other solution processed films. However, the record photocurrent for the state‐of‐the‐art CuWO_4_ thin films were reported for vapor phase deposited films with minimal grain boundaries and optimized annealing temperature [70].

**FIGURE 10:**
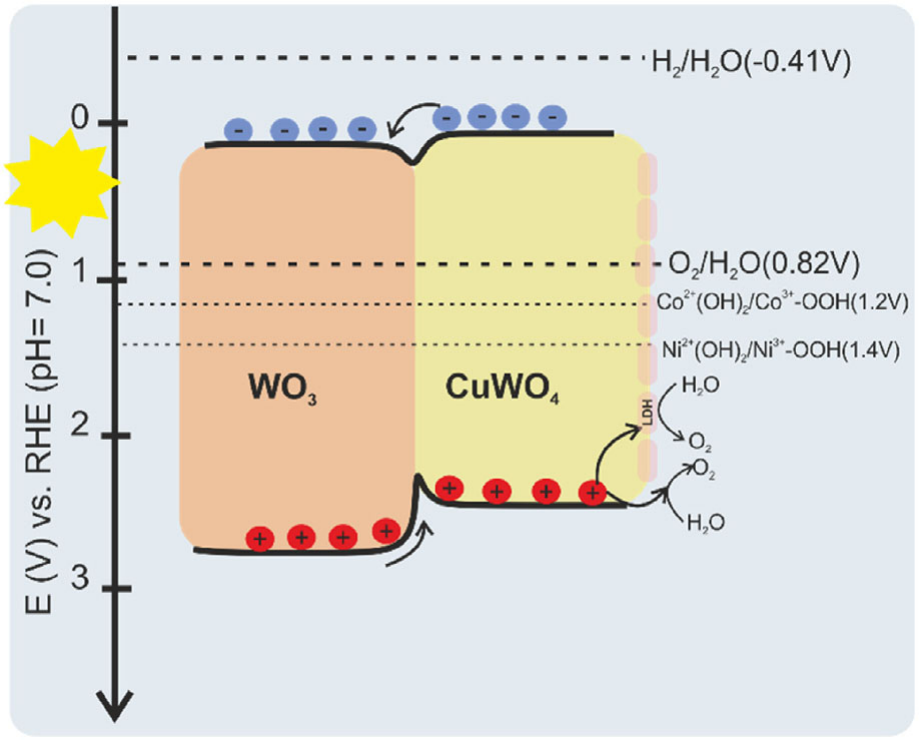
Schematics of the energy diagrams and charge transfer direction for CuWO_4_/WO_3_/LDH composite photoanode electrode.

In summary, the CuWO_4_/WO_3_ composite electrode investigated in this study most likely form a type II heterojunction as reported very recently by Escaliante et al. [20] were the photogenerated holes transfer from WO_3_ to the valence band of CuWO_4_. The holes then migrated to NiCo‐LDH, which cause the oxidation of the low‐valence M^2+^(Ni^2+^/Co^2+^) to high‐valence state of M^3+^(Ni^3+^/Co^3+^). This high‐valent metal ion serves as the actual active site, enabling the oxidation of water to oxygen. As a result, the LDH cocatalyst enhances electron transfer and promotes charge separation, which leads to the acceleration of OER kinetics and the improvement of the PEC properties for water splitting [38, 71]. On the other hand, the number and distribution of W rich microspheres control the ratios of CuWO_4_/WO_3_ and subsequently tune the heterojunction. Based on the reported band positions of CuWO_4_ and WO_3_ and own optical band and photocurrent onset measurements (Figure S8), and combining the redox transitions of the LDHs, the proposed band positions, and charge transfer is depicted in Figure 10.

## 4 Conclusion

In this study, the direct deposition of dandelion‐like CuWO_4_/WO_3_ heterojunction microarray onto the FTO substrate by using hydrothermal method was utilized. The growth mechanism of dandelion‐like microstructures was evaluated by controlling the hydrothermal reaction's parameters. During the initial stages of the hydrothermal reaction, the primary layer on FTO consisted of the CuWO_4_ phase, with an additional presence of excess W ions leading to the formation of the WO_3_ phase. As the reaction time increased, the surplus W precursor in the solution lead to the generation of spherical structures, which subsequently evolved into a dandelion‐like configuration. The photoelectrochemical activity of the photoanodes is strongly influenced by the morphology of the composite microarray on photoanodes. The sample with best PEC performance displayed a homogeneous distribution of microspheres on the substrate, characterized by neither complete coverage of the surface nor randomly scattered microspheres. This distribution facilitated the provision of enough accessible active sites on the surface to facilitate the photoelectrochemical reactions at the electrode/electrolyte interface. Further improvement of photoactivity of CuWO_4_/WO_3_ photoanode was achieved by integrating NiCo‐LDH co‐catalysts into the system for the first time. The CuWO_4_/WO_3_ photoanode adorned with the optimized NiCo‐LDH exhibits a photocurrent density of 0.17 mA cm^−2^ at 1.23 V versus RHE, a value twice as large as that of the pristine photoanode. The increased photocurrent density observed in CuWO_4_/WO_3_ electrodes can be attributed to the accelerated oxidation kinetics of both Ni^2+^ and Co^2+^ ions by photogenerated holes, which later involved in direct H_2_O oxidation. The promising PEC performance of modified photoanodes highlights the significant function of NiCo‐LDH in promoting accelerating charge transfer, facilitating effective separation of photogenerated charge carriers via consumption of accumulated holes on the electrode surface. The presented study demonstrated the potential to hydrothermal synthesis approach of more innovative tungsten‐based photoelectrodes, and development of binary and ternary LDHs as a novel oxygen evolution cocatalysts for improved performance in avoiding the use of precious metal catalysts in solar to chemical to energy conversion. Moreover, the performance of the current system can be further improved by fine tune of the film thickness and the post annealing temperature [70].

## Supporting Information

Additional supporting information can be found online in the Supporting Information section. SEM images, Raman Spectra, FTI‐IR, UV‐vis spectra, EDS mapping, XPS spectra and a table of performance comparison for representative solution processed CuWO_4_ systems are available. **Supporting Fig. S1:** SEM images of the CuWO_4_/WO_3_ showing the effect of synthesis time on the surface coverage: a) 4 h, b) 6 h, c) 8 h, d)10 h, e) 12 h, and f)14 h; g) Lateral and h) cross‐sectional view of the SEM images with light yellow lines showing the underlying dense film thickness; i) EDS spectra and elemental composition of CuWO_4_/WO_3_ composite film. **Supporting Fig. S2:** SEM images showing morphological variations of CuWO_4_/WO_3_ composite films prepared using different Cu:W ratios at 180°C for 8 h (a–c) and photocurrent response using Chopped light voltammetry (d). **Supporting Fig. S3:** Raman spectra of CuWO_4_/WO_3_ composite film prepared from precursor concentrations of 1 mM and 10 mM at 180°C and 8 h: a) flat area, b) sphere area and c) and d) the corresponding SEM images. **Supporting Fig. S4:** Raman spectra of CuWO_4_/WO_3_ composite film prepared from 2 mM precursor solution at 180°C with hydrothermal synthesis time of 4 h and 14 h: a) flat area, b) sphere area and c) and d) corresponding SEM images. **Supporting Fig. S5:** Raman spectra of CuWO_4_/WO_3_ composite film prepared hydrothermally for 8 h with temperatures at 140°C and 240°C. a) flat area, b) sphere area and c) and d) the corresponding SEM images. **Supporting Fig. S6:** FT‐IR spectra of NiCo‐LDH with various ratios of Ni and Co: (1:0), (2:1), (1:1), (1:2), (0:1). **Supporting Fig. S7:** SEM images of NiCo‐LDH decorated CuWO_4_/WO_3_ film and with corresponding element distribution mapping. **Supporting Fig. S8:** a) UV‐Vis absorption spectrum and b) Tauc plot of CuWO_4_/WO_3_ photoanode and NiCo‐LDH modified one. **Supporting Fig S9:** Chronoamperometry (i–t) curve of CuWO_4_/WO_3_ and CuWO_4_/WO_3_‐ NiCoLDH photoanodes at 1.6 V vs. RHE under chopped illumination in 0.1 M Na_2_SO_4_ (pH 7 PBS). **Supporting Fig. S10:** XPS spectra of CuWO_4_/WO_3_‐NiCo LDH before and after the PEC experiments a) Cu2p, b) W4f, c) Co2p and d) Ni2p. **Supporting Table S1:** Atomic percentage of elements NiCo‐LDH modified films. **Supporting Table S2:** PEC performance for CuWO_4_ thin films for the oxygen evolution reaction reported in the literature.

## Funding

This work was supported by Deutscher Akademischer Austauschdienst (57299294) and Deutsche Forschungsgemeinschaft (460244535).

## Conflicts of Interest

The authors declare no conflicts of interest.

## Supporting information

Supplementary Material

## Data Availability

The data that support the findings of this study are available from the corresponding author upon reasonable request.
