# Supplementary material for: Dandelion‐Like CuWO4/WO3 Composite Photoanode Employing Layered Double Hydroxide Catalysts for Enhanced Photoelectrochemical Water Oxidation
Source: ChemSusChem. 2025 Dec 14;19(1):e202502130. doi: 10.1002/cssc.202502130 (PMC12767566; doi:10.1002/cssc.202502130)
Supplement: Supplementary file 1 — Supplementary Material [file CSSC-19-e202502130-s001.pdf]

Supporting Information

Dandelion-Like $CuWO_4/WO_3$ Composite Photoanode Employing Layered Double Hydroxide Catalysts for enhanced Photoelectrochemical Water Oxidation

Sima Nouhi, Michael Wark, Dereje Hailu Taffa*

Institute of Chemistry, Chemical Technology 1, Carl von Ossietzky University of Oldenburg,

Carl-von-Ossietzky-Str. 9-11, 26129 Oldenburg, Germany

E-mail: dereje.hailu.taffa@uni-oldenburg.de

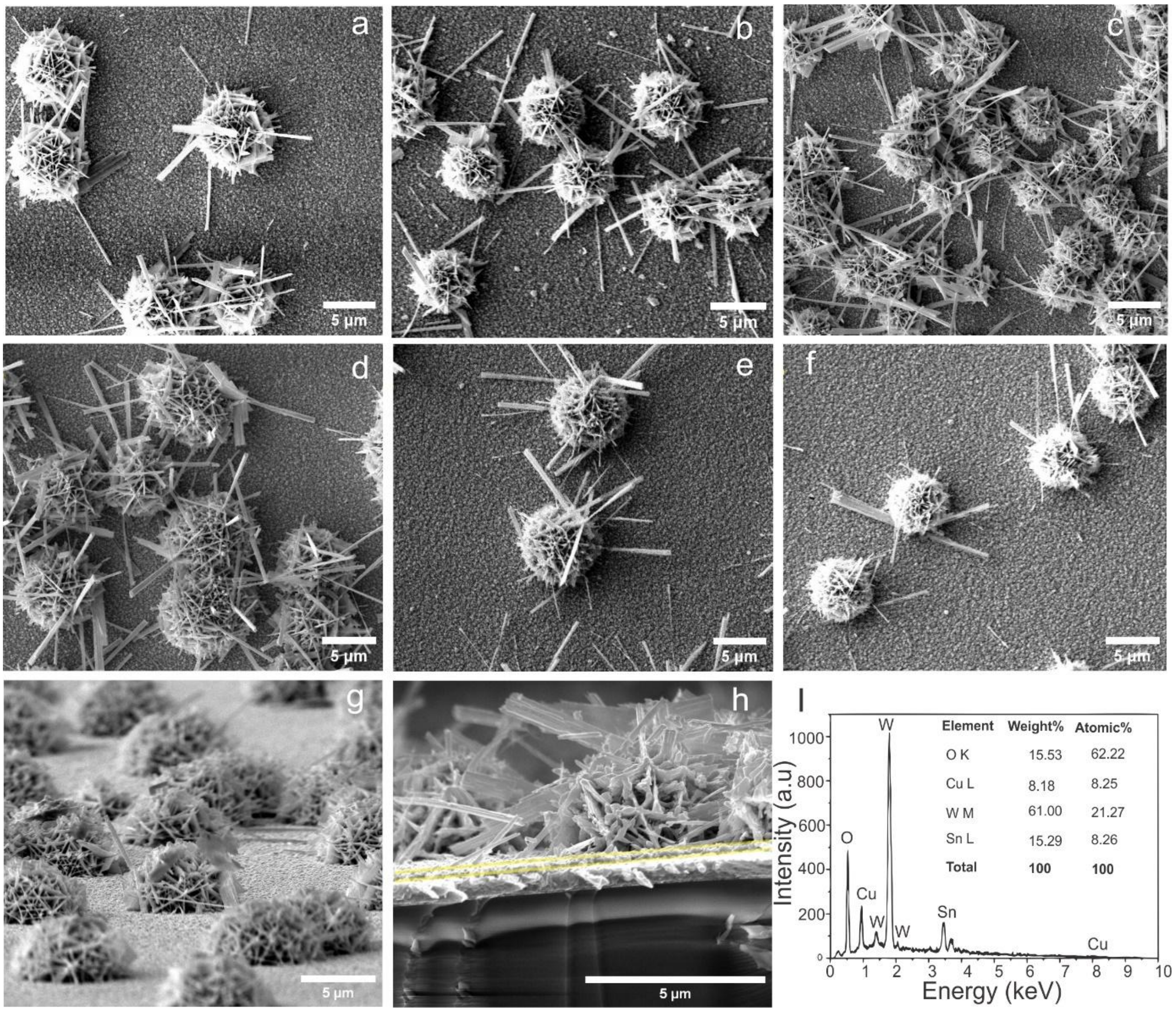

Fig. S1. SEM images of the $CuWO_4/WO_3$ showing the effect of synthesis time on the surface coverage: a) 4h, b) 6h, c) 8h, d)10h, e)12h, and f)14h; g) Lateral and h) cross-sectional view of the SEM images with light yellow lines showing the underlying dense film thickness; i) EDS spectra and elemental composition of $CuWO_4/WO_3$ composite film.

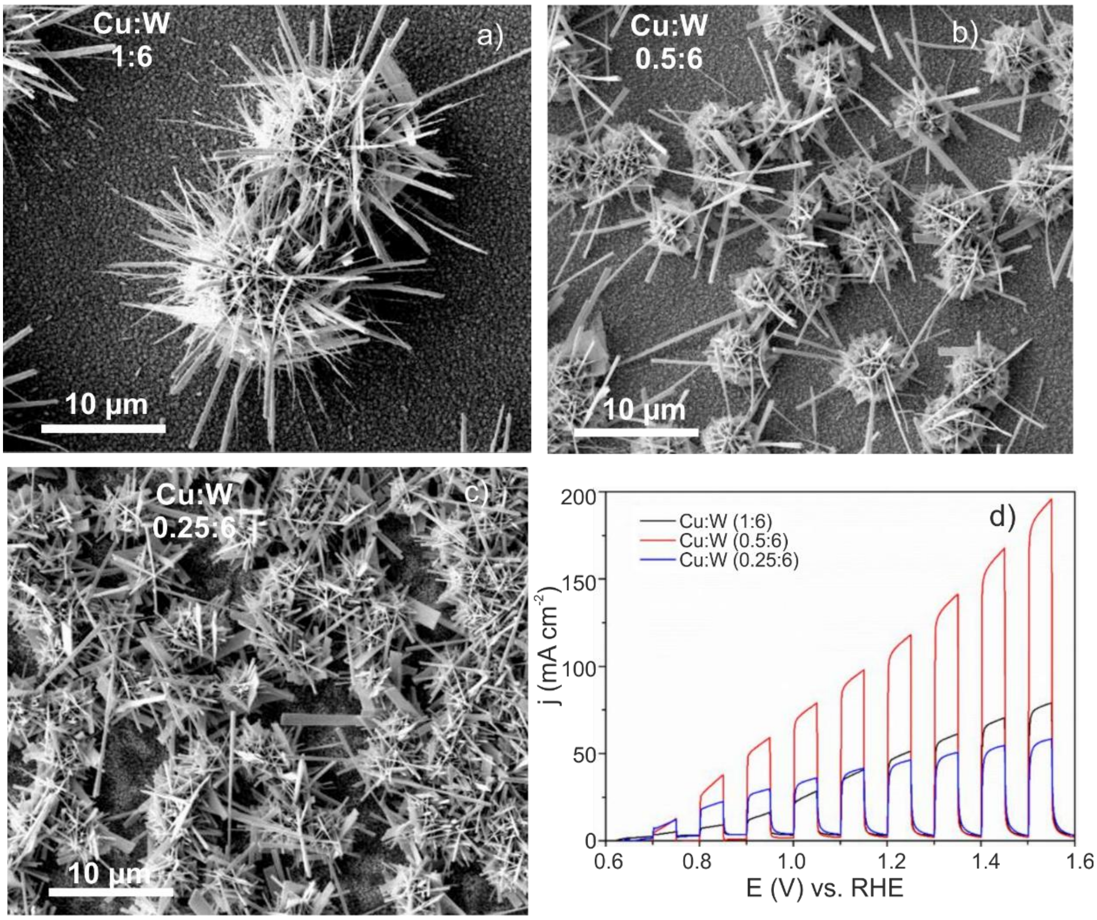

Fig. S2. SEM images showing morphological variations of $CuWO_4/WO_3$ composite films prepared using different Cu:W ratios at 180˚C for 8h (a-c) and photocurrent response using Chopped light voltammetry (d).

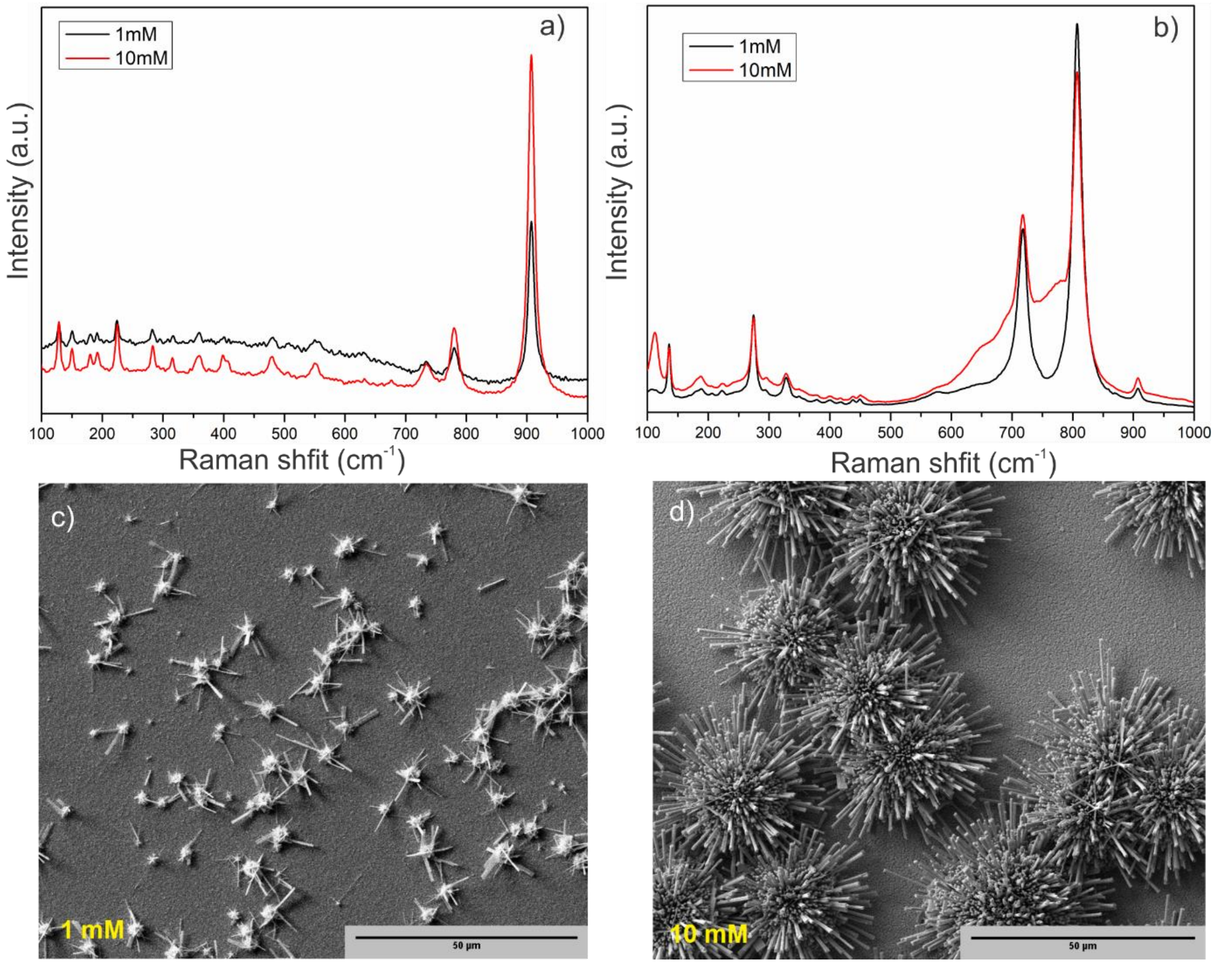

Fig. S3. Raman spectra of $CuWO_4/WO_3$ composite film prepared from precursor concentrations of 1mM and 10mM at 180 °C and 8h: a) flat area, b) sphere area and c) and d) the corresponding SEM images.

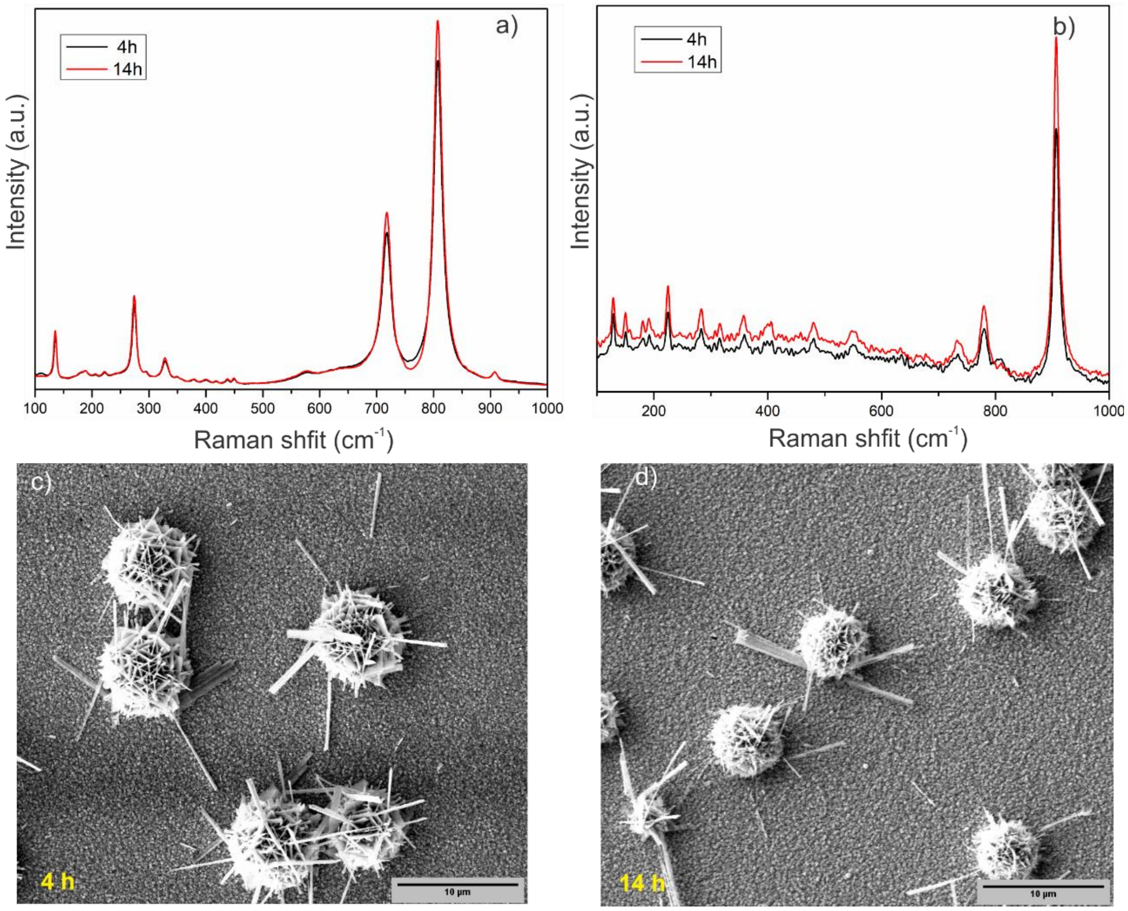

Fig. S4. Raman spectra of $CuWO_4/WO_3$ composite film prepared from 2mM precursor solution at 180 °C with hydrothermal synthesis time of 4h and 14h: a) flat area, b) sphere area and c) and d) corresponding SEM images.

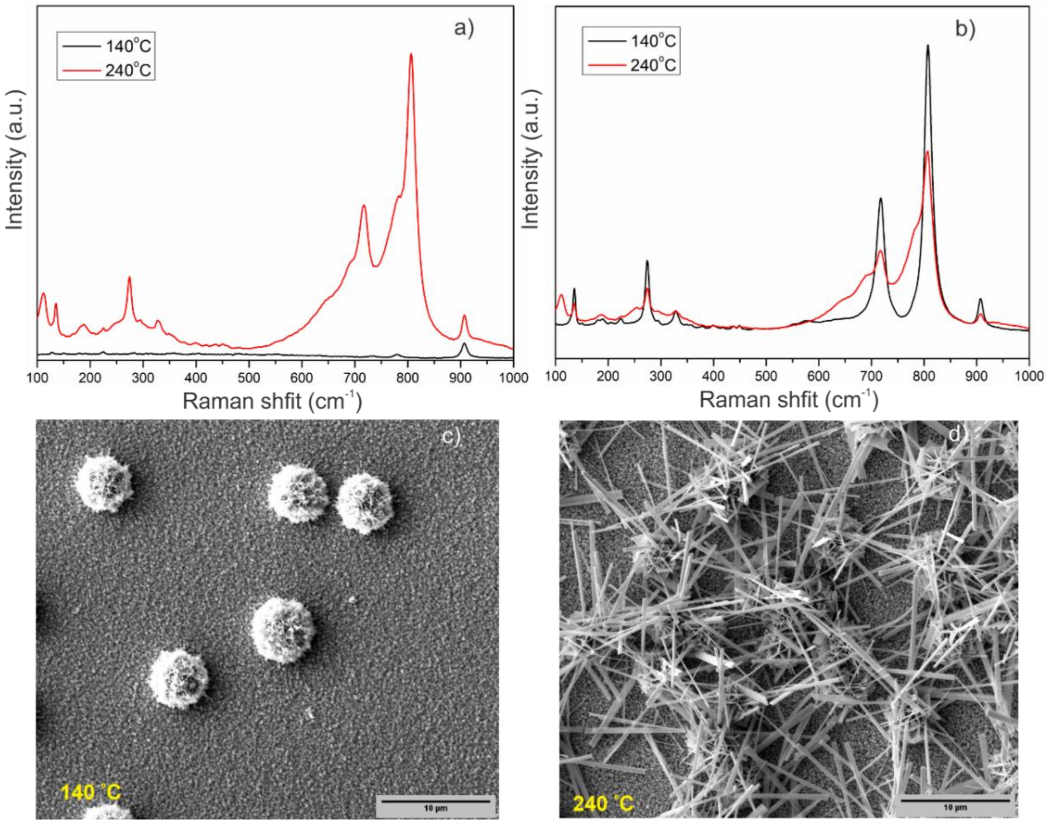

Fig. S5. Raman spectra of $CuWO_4/WO_3$ composite film prepared hydrothermally for 8h with temperatures at 140˚C and 240˚C. a) flat area, b) sphere area and c) and d) the corresponding SEM images.

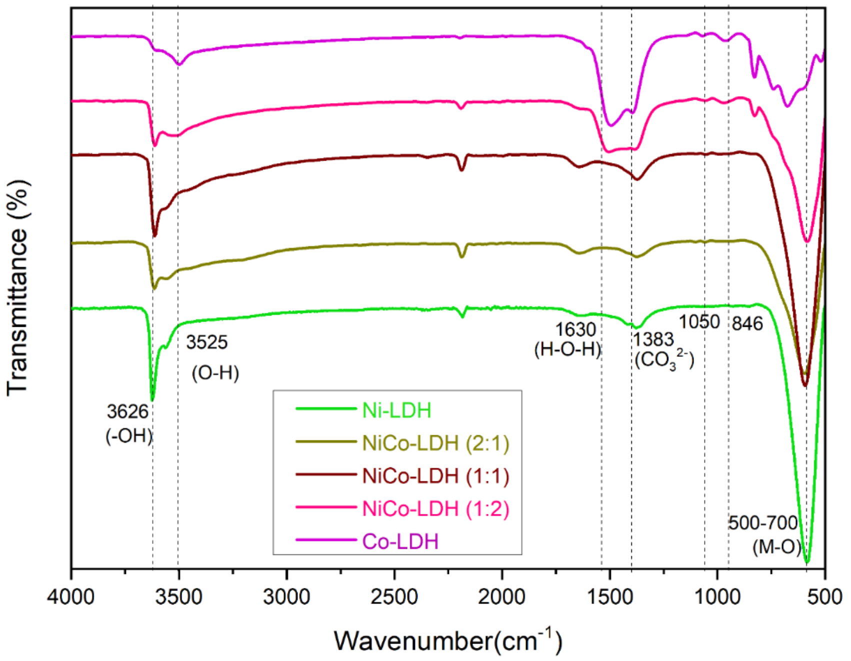

Fig. S6. FT-IR spectra of NiCo-LDH with various ratios of Ni and Co: (1:0), (2:1), (1:1), (1:2), (0:1).

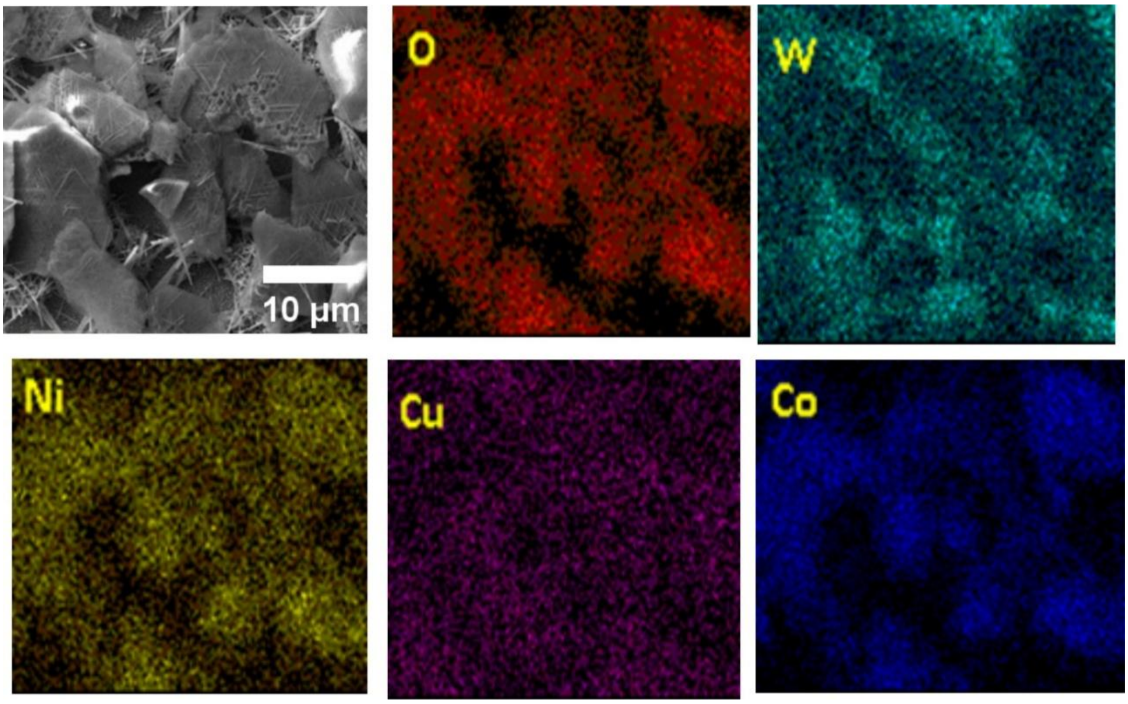

Fig. S7. SEM images of NiCo-LDH decorated $CuWO_4/WO_3$ film and with corresponding element distribution mapping.

Table S1. Atomic percentage of elements NiCo-LDH modified films

Element	(XPS) At%	(EDX) At%
O	32.01	84.08
Co	26.16	5.28
Ni	13.34	2.95
Cu	4.83	1.35
W	23.66	6.78
Total	100.00	100.00

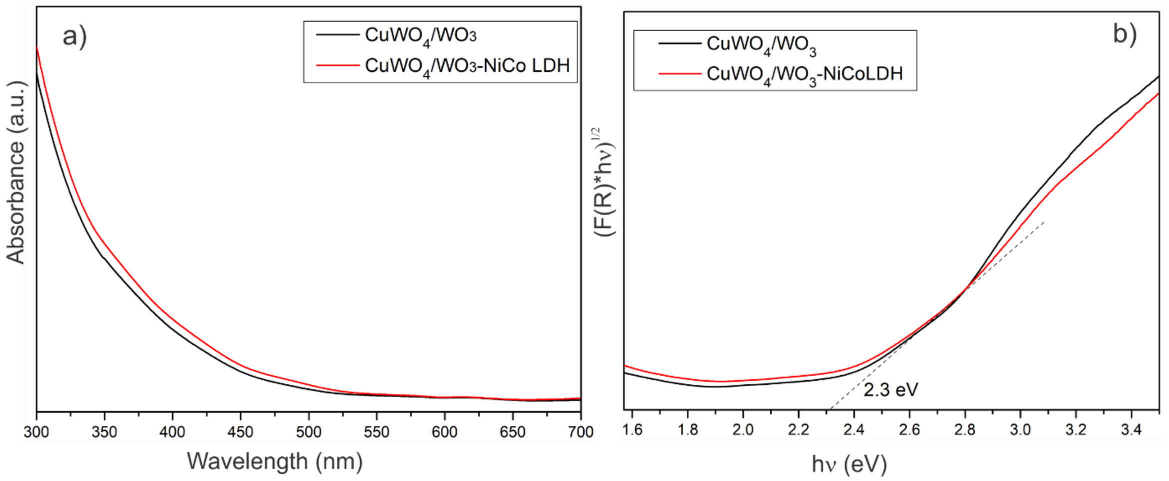

Fig. S8. a) UV-Vis absorption spectrum and b) Tauc plot of $CuWO_4/WO_3$ photoanode and NiCo-LDH modified one.

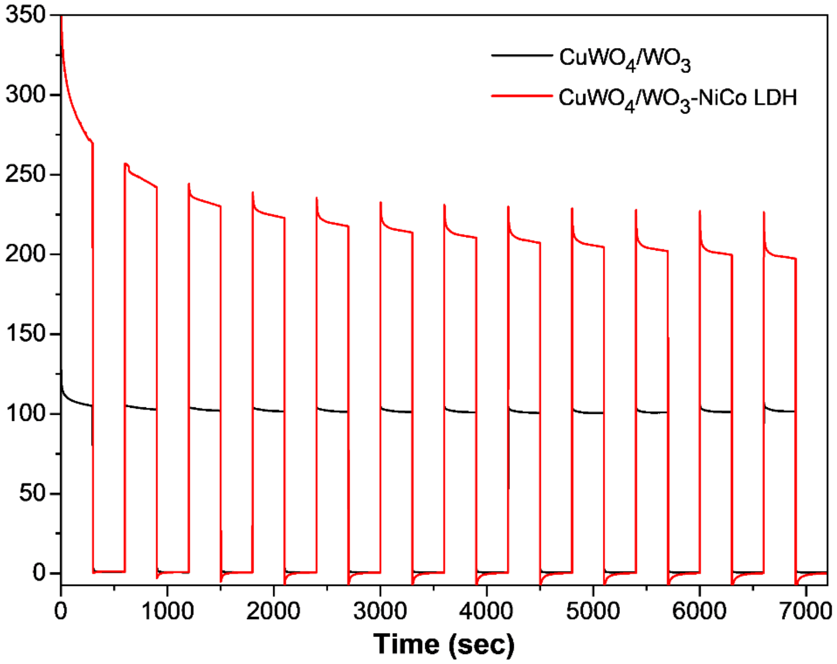

Fig S9. Chronoamperometry (i–t) curve of $CuWO_4/WO_3$ and $CuWO_4/WO_3$- NiCoLDH photoanodes at 1.6 V vs. RHE under chopped illumination in 0.1 M Na2SO4 (pH 7 PBS).

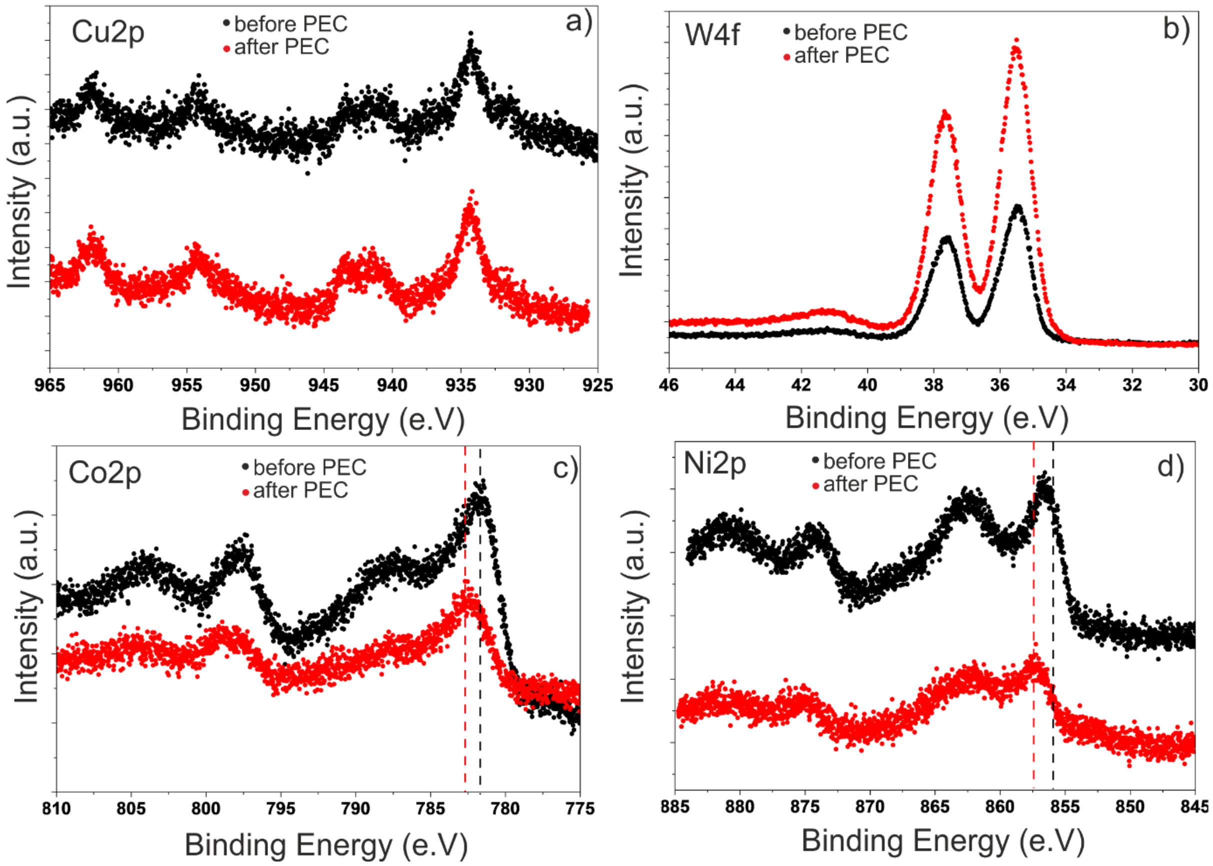

Fig. S10. XPS spectra of $CuWO_4/WO_3$-NiCo LDH before and after the PEC experiments a) Cu2p, b) W4f, c) Co2p and d) Ni2p

Table S2. PEC performance for $CuWO_4$ thin films for the oxygen evolution reaction reported in the literature.

Synthesis method	Modified with	Electrolyte	$J_{ph}^{a,b}$ *(mA/cm^2) at 1.23 V vs. RHE	Ref.
Spray pyrolysis	Au	0.1 M PBS pH=7	J_{ph}^a = 0.03<br>J_{ph}^b = 0.1	[2]
Electrodeposition	Mo (doping)	0.1 M PBS pH=7	J_{ph}^a = 0.07<br>J_{ph}^b = 0.16	[7]
Spin coating	__	0.1 M PBS pH=7	J_{ph}^a = 0.15	[12]
Electrodeposition	__	0.1 M PBS pH=7	J_{ph}^a = 0.16	[13]
Hydrothermal	H_2 treatment	0.1 M Na_2SO_4 solution (pH = 6.8)	J_{ph}^a = 003<br>J_{ph}^b = 0.19	[25]
Spray pyrolysis	Fe (doping)	0.1 M PBS pH=7	J_{ph}^a = 0.19<br>J_{ph}^b = 0.30	[27]

HT/Drop casting	Ni-P_i	0.1 M PBS pH=7	J_{ph}^a = 0.3<br>J_{ph}^b = 0.5	[32]
Thermal solid-state reaction	Co-P_i	0.1 M PBS pH=7	J_{ph}^a = 0.34<br>J_{ph}^b = 0.42	[65]
Hydrothermal	NiCo-LDH	0.1 M Na_2SO_4 PBS solution (pH = 7)	J_{ph}^a = 0.1<br>J_{ph}^b = 0.17	This work

*J_{ph} represents the photocurrent density. PBS represents phosphate buffer solution. HT represents hydrothermal. J_{ph}^a is assigned to the J value of pristine $CuWO_4$, and J_{ph}^b is assigned to the J value of the modified $CuWO_4$ photoanode.
